# Complementary Food Supplements Fill Energy and Protein Gaps among Children with Dietary Inadequacy in a Complementary Feeding Trial in Rural Bangladesh

**DOI:** 10.1016/j.tjnut.2024.12.001

**Published:** 2024-12-09

**Authors:** Monica M Pasqualino, Rebecca K Campbell, Kristen M Hurley, Lee S-F Wu, Abu Ahmed Shamim, Saijuddin Shaikh, Saskia de Pee, Parul Christian

**Affiliations:** 1 Department of International Health, Johns Hopkins Bloomberg School of Public Health, Baltimore, MD, United States; 2 Division of Epidemiology and Biostatistics, University of Illinois Chicago, Chicago, IL, United States; 3 Center for Noncommunicable Diseases and Nutrition, BRAC James P Grant of School of Public Health, BRAC University, Mohakhali, Dhaka, Bangladesh; 4 The JiVitA Project, Gaibandha, Bangladesh; 5 World Food Programme, Rome, Italy

**Keywords:** complementary feeding, supplementation, energy, macronutrients, South Asia

## Abstract

**Background:**

Few studies have evaluated the dietary impact of complementary food supplements (CFSs) designed to deliver macro- and micronutrients to children at risk for undernutrition. In a randomized controlled trial in rural Bangladesh, we previously reported that CFSs increased children’s micronutrient adequacy.

**Objectives:**

To longitudinally characterize energy and macronutrient intakes and inadequacies and evaluate the extent to which CFSs fill intake gaps.

**Methods:**

Children were enrolled at 6 mo and received 1 of 4 CFSs plus caregiver nutrition counseling or counseling alone for 1 y. A semi-quantitative diet questionnaire was administered at 6, 9, 12, 15, 18, and 24 mo. Energy and macronutrient intakes were estimated by age and arm; protein adequacy was adjusted for protein quality and infection. We estimated the proportion meeting intake requirements set by FAO and the Institute of Medicine and compared group-wise differences using log binomial regression models with generalized estimating equations. We used multivariate analysis of variance models to evaluate if CFSs substituted home foods.

**Results:**

Across groups, most children did not meet energy or protein requirements at enrollment (74.6%–81.3% and 77.4%–79.2%, respectively). Estimated energy and macronutrient intakes from home foods increased from 6 to 24 mo. Energy inadequacy was lower in the supplemented groups compared with the control at all ages (e.g. 10.5%–13.8% compared with 31.4% at 18 mo). In the control group, protein inadequacy dropped from 78.4% at 6 mo to 8.3% at 9 mo to 2.8% by 18 mo; adjusted protein estimates were 25.1% at 9 mo and 7.0% at 18 mo. Protein inadequacy was the highest in the control group at all timepoints. CFSs did not substitute home foods.

**Conclusions:**

CFSs can significantly bridge energy and protein intake gaps. With earlier trial findings that CFSs filled micronutrient gaps and improved growth, these findings strengthen evidence supporting using CFSs for improved health outcomes.

This trial was registered at clinicaltrials.gov with registration number NCT01562379 (https://clinicaltrials.gov/ct2/show/NCT01562379).

## Introduction

The complementary feeding period from 6 to 23 mo of age is a critical time for growth and development. Linear growth faltering during this time is common, leading to a high prevalence of stunting by 24 mo of age in low- and middle-income countries (LMICs) [1]. As infants and young children age, breastmilk intake decreases and the energy and nutrient needs from complementary foods increase [2]. Meeting these needs is challenging in low-resource settings where access to affordable, diverse, and energy- and nutrient-dense foods is limited [3,4]. Inadequate energy and nutrient intakes early in life contribute to an increased risk of growth faltering and infection, as well as to impaired cognitive development and a higher risk for noncommunicable disease in adulthood [5].

Various formulations of complementary food supplements (CFSs) have been developed to fill energy and nutrient gaps among young children at risk for undernutrition [6]. Among these, lipid-based nutrient supplements (LNS), which provide energy, protein, essential fatty acids, and multiple micronutrients in a food base, are available in a range of daily portion sizes intended for the prevention or treatment of undernutrition [7]. Preventive small-quantity LNS, providing 118 kcal and 2.6 g of protein, is a cost-effective intervention that has been shown in meta- and individual participant analyses to have a beneficial impact on child growth, anemia, and mortality [[8], [9], [10], [11]]. A community-based, cluster randomized-controlled trial (JiViTA-4), which contributed to meta-analyses on LNS, was implemented in rural Bangladesh to test the effect of 4 types of CFSs provided daily (2 locally produced and 1 commercially produced LNS, and 1 fortified blended wheat-soy formulation) compared with a control arm that received no supplementation from 6 to 18 mo of age; all arms received child feeding counseling (CFC). The primary outcomes were length-for-age z-score (LAZ) and stunting, with the trial finding a decline in linear growth deceleration and reduction in stunting prevalence in the supplemented groups compared with the control group at 18 mo of age [12]. Dietary intakes were secondary outcomes of the trial. Previously reported findings from the trial on micronutrient intakes showed that supplementation did not replace home-based diets; estimated micronutrient intakes from home foods were inadequate overall, and the CFSs provided filled intake gaps for many micronutrients, including iron, zinc, vitamin A, and B-vitamins [13].

Overall, few studies have quantified energy, protein, and other nutrient intakes during the complementary feeding period in LMICs. Precise and accurate dietary data are needed to characterize the typical diet and measure the specific energy and macronutrient gaps of a population, especially because intake gaps often vary considerably across contexts [14]. Additionally, it is important to know if replacement of home-based complementary foods occurs because of supplementation.

Using dietary data collected through a semistructured 24-h diet questionnaire under the JiVitA-4 trial, we aimed to characterize, longitudinally from 6 to 18 mo of age, 1) change in typical energy and macronutrient intakes from home-based complementary diets as children aged, 2) intake adequacy of energy and protein, unadjusted and adjusted for protein quality and infection, 3) the extent to which each of the 4 CFSs provided daily filled energy and unadjusted and adjusted protein intake gaps, and 4) whether the CFSs led to any substitution of energy and macronutrients from home-based foods.

## Methods

### Setting and study population

The trial was implemented from September 2012 to May 2014 at the JiVitA research site in the Gaibandha and Rangpur districts in northwestern Bangladesh. The study area, which was divided into 596 clusters, is densely populated with a majority Muslim population and is considered representative of national maternal and child nutritional status [15]. Data from the present trial were consistent with those from the 2014 Demographic and Health Survey (DHS). For example, the prevalence of stunting was ∼20% among 6-mo-olds in the present trial and among 6–8-mo olds in the DHS; almost all children were breastfed at 6 mo and continued breastfeeding at 12 mo in both this study and nationally [16].

### Study design and interventions

The details of the trial are published elsewhere [12]. Briefly, the trial had a 5-arm, cluster-randomized controlled design to test the effect of 4 types of CFSs on linear growth and rates of stunting and wasting among children from 6 to 18 mo of age. Clusters were allocated to 1 of the 4 CFS groups or the control group (no food supplement). Children (n = 5449) were enrolled into the trial at 6 mo of age. The CFS products included 2 locally produced LNS that were ready-to-use, 1 with chickpeas (CP) as the main ingredient and 1 with a mix of rice and lentils (RL) (development of these products have been detailed elsewhere); enhanced wheat-soy blend (WSB++); a fortified blended food supplied by the World Food Programme; and Plumpy’doz (PD), a commercially produced LNS that contains peanut paste, vegetable oil, and milk powder (Nutriset) [17]. The CFS products were isocaloric and approximately balanced in micronutrient content. They were delivered to children from 6 to 11 mo of age daily in a single portion/sachet providing 125 kcal and 2.6 g protein (small quantity) and from 12 to 18 mo of age 250 kcal and 5.2 g (medium quantity) for a total duration of 1 y of supplementation. The CFSs were delivered weekly by local female study workers as per random allocation during home visits accompanied by counseling and monitoring of adherence, and replenished at each visit. Study workers provided instructions to caregivers on how to prepare and feed the CFSs to children, including feeding the supplements every day in addition to usual foods in the diet and breastmilk, and counseled them to not share the supplements with other family members. CFC was delivered approximately monthly to caregivers in all arms through age-specific messages on appropriate infant and young child feeding and hygiene practices on the basis of the modules developed by Alive and Thrive for use in rural Bangladesh [18].

### Sample size

The sample size was determined according to the primary aims of the trial, which were gains in LAZ and reduced rates of stunting at 18 mo in the supplemented groups compared with the control group and noninferiority of LAZ and stunting of groups receiving CP, RL, and WSB++ compared with the group receiving PD. Assumptions of α = 0.05, β = 0.10, and a 5% loss to follow-up were applied. Sample size calculations were adjusted for the cluster-randomized design with a design effect of 1.5, and allowed for multiple comparisons between groups. This resulted in a sample size of 5320 children to be enrolled in the trial. This large sample size allowed for robust examinations of dietary intakes between groups as reported here, which was a secondary analysis from the trial.

### Data collection

Home-based interviews were scheduled at 6 (enrollment), 9, 12, 15, and 18 mo to collect data on child characteristics, including dietary intake, breastfeeding status, and anthropometry. A post-supplementation follow-up visit was scheduled at 24 mo to estimate long-term effects. Household and parental characteristics were assessed at the enrollment visit [12]. To measure dietary intake, trained interviewers administered a semistructured quantitative 24-h diet questionnaire at each visit that included a list of 29 solid, semisolid, and liquid food items that had been identified as commonly consumed foods by children of similar age in this setting using 7-d food-frequency data previously collected. The food list included individual foods (e.g. banana), cooked recipes, and food categories (e.g. fish and leafy vegetables). A qualitative study was undertaken to confirm the food list, develop standard recipes, generate weight measures in grams for each item with standard measurements, and identify market-purchased foods commonly fed to young children [19].

For each item, the interviewer asked, “From yesterday morning to today morning, has the child been fed [the item]?” If the caregiver responded “yes,” the interviewer asked how much of the item was fed and recorded the quantity and unit of measurement using standard measures (e.g. spoons, bowls, and glasses); interviewers carried the standard measures with them to show caregivers during the interview process. After completing the prespecified list, the interviewer asked if the child had eaten any additional foods. Up to 6 additional items were recorded and subsequently coded and included in analyses. We monitored how caregivers reported additional items at each interview to ensure foods weren’t limited because of space limitations in the questionnaire. Breastfeeding status was assessed at each visit by asking the caregiver if the child was currently being breastfed. If yes, she was asked how many times in the last 24 h the child had been breastfed, with response options of 1–10, 11–20, or ≥21 times.

For CFS consumption, study workers visited households twice a week to assess adherence to the CFSs in the supplemented groups by counting sachets and asking caregivers to recall the amount offered, consumed, left over, or shared each day. Weekly morbidity data were collected in all groups.

### Ethical approval

Study protocols were approved by the Institutional Review Board of the Johns Hopkins Bloomberg School of Public Health and Ethical Review Committee of the International Center for Diarrhoeal Disease Research. Written parental consent was obtained at enrollment. The trial was registered at clinicaltrials.gov as NCT01562379. The present study reports on secondary dietary intake data that were not included in the trial registration.

### Statistical analysis

To convert food intake data to energy and nutrient intakes, we used food composition tables from Bangladesh and other South Asian countries [[20], [21], [22], [23], [24], [25], [26]]. In each group, we summed the energy and macronutrient intakes across all food items consumed by each child at each interview to estimate intakes from home-based complementary foods. Within the supplemented groups, to estimate each child’s energy and nutrient intake from the CFSs, we calculated the percentage of the supplement dose reportedly consumed on the day before each interview, i.e. during the period of time referred to in reported intake in the last 24 h. Because we did not directly measure breastmilk intake, and continuation of breastfeeding through 18 mo of age was almost universal across groups, we assumed age-specific mean breastmilk intake with typical energy and macronutrient content for all children using age ranges (i.e. 6–8 mo, 9–11 mo, and 12–23 mo) for each relevant timepoint based on published values [4,27,28]. Sensitivity analyses were conducted assuming that breastmilk intake was ±5, 10, 15, and 20% compared with the referenced values.

We explored energy and macronutrient intakes for outlying values and omitted intakes of implausibly high energy, as detailed elsewhere [13]. From each diet recall interview, we estimated energy and macronutrient intakes from complementary foods prepared in the home. To evaluate intake inadequacy by age and by group, we estimated the proportion of children with total estimated intakes (from home-based foods and assumed breastmilk) below dietary recommendations. We calculated total energy requirements on the basis of FAO guidelines, which are sex- , weight-, and age-specific [29]. For protein, we referred to the following values set by the Institute of Medicine (IOM): 1.52 g/kg/d through 6 mo, 1.0 g/kg/d from 7 to 12 mo, and 0.87 g/kg/d from 1 to 3 y [30]. For energy and protein, we used the mean control group weight at each age to set a standard requirement across children and groups. For fat, we used the adequate intake (AI) value from the IOM for 9 mo (30 g/d); no values are defined for 12–18 mo of age. For carbohydrates, we referred to the AI for 9 mo (95 g/d) and Recommended Daily Allowance for 12–18 mo (130 g/d) [30]. We compared the proportions meeting intake recommendations in the supplemented compared with unsupplemented groups using log binomial regression models with generalized estimating equations (GEE) to account for clustering.

We adjusted protein intakes using the Digestible Indispensable Amino Acid Score (DIAAS) method to estimate available protein intakes in all children except for those receiving PD as information is not available to estimate DIAAS for this commercially produced product [31]. Data on amino acids were obtained from food composition tables from Bangladesh and other countries, and digestibility factors from FAO and the literature [[20], [21], [22], [23], [24], [25], [26],^32^,^33^]. We further adjusted protein intake for inadequate energy intake and for frequent infection by assuming 10% additional protein requirement because of dietary energy deficiency plus 10% increase in protein requirement during each day of illness, assuming 5.3 episodes per year with each episode lasting 7 d, or 11.1% increase in protein requirement relative to the estimated average requirement [34].

We assessed if intake of the CFSs resulted in the substitution of home foods by comparing estimated mean energy and nutrient intakes from home-based complementary foods in the supplemented groups to the control group using multivariate analysis of variance models (MANOVA) for each age. Models included macro- and micronutrients, adjusted for energy intake, as dependent variables and treatment group as the independent variable, and were analyzed separately for each age. When MANOVA models were significant, we used linear regressions models with GEE for energy and each nutrient to test group-wise differences. All analyses were conducted in Stata, version 14.1 (StataCorp). Here, we report on the substitution of macronutrient intakes; details on the substitution of micronutrient intakes (i.e. calcium, iron, magnesium, phosphorus, potassium, zinc, copper; vitamins A, B6, D, E, C; thiamin, riboflavin, niacin, folate) are presented elsewhere [13].

## Results

Of the 5449 children enrolled in the trial, almost all (93%–98.8%) had available dietary intake data at each interview and 86.3% had data available for all interviews [13]. Groups were comparable on most child, parental, and household characteristics at enrollment (6 mo) (Supplemental Table 1) [12]. Across groups, 50% of children were male, 26% were stunted, 76% had mothers with some education, and 50% lived in households experiencing food insecurity at enrollment [12]. Almost all (97%) children across groups were still breastfeeding at the 18-mo interview [27]. Adherence to the CFSs was high and did not differ between the supplemented groups [12].

Estimated intakes of energy and macronutrients from home-based complementary foods increased from 6 to 24 mo of age in the unsupplemented control group (Table 1). Across groups, estimated total energy and macronutrient intakes summed across intakes from home complementary foods, CFSs [in the supplemented groups], and breastmilk increased as children aged (Supplemental Table 2).

**TABLE 1:** Energy and macronutrient intakes from home complementary foods among children enrolled in the unsupplemented control arm of a complementary food supplementation trial in Bangladesh, by age.

	6 mo		9 mo		12 mo		15 mo		18 mo		24 mo	
	*n*	Median (25th, 75th pctl)	*n*	Median (25th, 75th pctl)	*n*	Median (25th, 75th pctl)	*n*	Median (25th, 75th pctl)	*n*	Median (25th, 75th pctl)	*n*	Median (25th, 75th pctl)
Energy (kcal/d)	1387	47 (9, 110)	1337	199 (109, 319)	1328	311 (185, 460)	1308	394 (249, 589)	1271	485 (316, 704)	1237	618 (414, 897)
Protein (g/d)	1387	0.7 (0.1, 2.1)	1337	5.0 (2.3, 8.5)	1328	7.8 (4.3, 12.7)	1308	10.5 (5.8, 16.3)	1271	12.9 (7.7, 19.4)	1237	16.0 (10.2, 23.7)
Percent calories from protein	1108	6.8 (5.2, 10.4)	1294	9.2 (7.1, 12.7)	1315	9.9 (7.8, 12.7)	1292	10.0 (8.1, 12.7)	1266	10.4 (8.3, 12.8)	1234	10.1 (8.4, 12.1)
Fat (g/d)	1387	1.0 (0.0, 2.7)	1337	4.7 (2.0, 8.6)	1328	6.8 (3.5, 11.8)	1308	9.3 (4.7, 15.3)	1271	12.0 (6.4, 18.4)	1237	13.6 (8.3, 21.3)
Percent calories from fat	1108	25.1 (11.1, 35.4)	1294	22.7 (14.5, 31.6)	1315	21.2 (14.6, 28.8)	1292	22.1 (14.5, 29.7)	1266	22.9 (16.0, 30.6)	1234	20.5 (14.7, 26.9)
Carbohydrates (g/d)	1108	10.4 (4.3, 21.6)	1294	32.5 (18.0, 52.5)	1315	51.4 (30.1, 79.3)	1292	65.6 (40.3, 97.8)	1266	78.7 (49.2, 115.3)	1234	105.1 (69.4, 153.3)
Percent calories from carbohydrates	1108	67.1 (54.5, 83.0)	1294	68.2 (55.9, 77.5)	1315	68.9 (59.0, 76.8)	1292	67.7 (58.2, 76.8)	1266	66.7 (57.5, 74.8)	1234	69.4 (61.0, 76.3)

At enrollment (6 mo), a majority of children (74.6%–81.3%) across arms did not meet the intake requirements for energy (Table 2). In the unsupplemented control arm, as children aged, energy intake inadequacy decreased but remained relatively high (31.4%) by the end of the intervention at 18 mo. From 9 to 18 mo, the prevalence of inadequate energy intake was lower in all supplemented groups relative to the control group. For example, at 9 mo, the proportion not meeting the intake requirement was 48.7% in the control group compared with 14.0%–16.0% in the supplemented groups.

**TABLE 2:** Prevalence of inadequate energy and protein intakes by age and supplement group among children enrolled in a complementary food supplementation trial in Bangladesh^1^.

Age and Group	Energy						Protein			
	EAR, kcal/d^2^	Inadequate intake, n (%)	Chi^2^ pvalue^3^	EAR, g/d^2^	Unadjusted		DIAAS-adjusted^4^		DIAAS, energy and infection adjusted^4^^,^^5^	
					Inadequate intake, n (%)	Chi^2^ pvalue^3^	Inadequate intake, n (%)	Chi^2^ pvalue^3^	Inadequate intake, n (%)	Chi^2^pvalue^3^
6 mo (presupplementation)	520.5		0.0005	10.3		0.9619		0.9007		0.7276
CFC-only		1035 (74.6)			859 (78.4)		903 (82.5)		958 (87.5)	
Plumpy’doz		1181 (81.3)			786 (78.1)		819 (81.3)		881 (87.5)	
Chickpea		646 (78.7)			477 (78.2)		490 (80.3)		517 (84.8)	
Rice-lentil		647 (80.5)			442 (79.2)		456 (81.7)		484 (86.7)	
WSB++		670 (80.6)			453 (77.4)		480 (82.1)		511 (87.4)	
9 mo	573.5		<0.0001	7.4		<0.0001		<0.0001		<0.0001
CFC-only		651 (48.7)			107 (8.3)		159 (12.3)		324 (25.1)	
Plumpy’doz		226 (16.2)			4 (0.3)		-		-	
Chickpea		115 (14.7)			3 (0.4)		3 (0.4		8 (1.0)	
Rice-lentil		109 (14.0)			7 (0.9)		12 (1.5)		22 (2.8)	
WSB++		125 (16.0)			3 (0.4)		5 (0.6)		11 (1.4)	
18 mo	704.1		<0.0001	7.5		0.0005		<0.0001		<0.0001
CFC-only		399 (31.4)			35 (2.8)		59 (4.7)		88 (7.0)	
Plumpy’doz		140 (10.5)			7 (0.5)		-		-	
Chickpea		96 (12.7)			5 (0.7)		5 (0.7)		13 (1.7)	
Rice-lentil		104 (13.8)			6 (0.8)		9 (1.2)		14 (1.9)	
WSB++		84 (11.1)			8 (1.1)		10 (1.3)		19 (2.5)	
24 mo (postsupplementation)	778.0		0.0062	8.3		0.6325		0.2476		0.4476
CFC-only		335 (27.1)			34 (2.8)		57 (4.6)		86 (7.0)	
Plumpy’doz		298 (23.9)			27 (2.2)		43 (3.5)		65 (5.2)	
Chickpea		175 (25.0)			15 (2.2)		22 (3.2)		43 (6.2)	
Rice-lentil		191 (26.9)			14 (2.0)		25 (3.5)		42 (5.9)	
WSB++		145 (19.7)			14 (1.9)		21 (2.9)		41 (5.6)	

Abbreviations: CFC, child feeding counseling; DIAAS, Digestible Indispensable Amino Acid Score; EAR, Estimated Average Requirement; WSB++, wheat-soy blend.

1 Energy and protein intakes include reported complementary food intake from a 24-h recall, reported daily supplement intake, and age-specific estimated breast-milk intake. From 6 to 11 mo, the supplements were delivered in small quantities of 125 kcal/d and 2.6 g of protein. From 12 to 18 mo, the supplements were delivered in medium quantities of 250 kcal/d and 5.2 g of protein. It was not possible to estimate adjusted protein intake for the group receiving PD from 9 to 18 mo because information is not available to estimate the DIAAS for this commercially produced product.

2 Estimated using mean weight of control group infants and children at each age timepoint.

3 Chi^2^*P* values are from log binomial regression models with generalized estimating equations to allow for clustering of observations within sectors.

4 Using the Digestible Indispensable Amino Acid Score to adjust for protein quality.

5 EAR adjusted for DIAAS plus inadequate energy intake and infections assuming 10% additional protein requirement because of dietary energy deficiency, plus 10% increase in protein requirement during each day of illness, 7 d per episode, 5.3 episodes per year, or 11.1% increase in protein requirement relative to the EAR.

Protein intake inadequacy was also high at enrollment across arms (77.4%–79.2%, unadjusted; 84.8%–87.5%, adjusted for protein quality and infection, Table 2). In the unsupplemented control arm, inadequacy decreased dramatically with protein intake dropping from 78.4% at 6 mo to 8.3% at 9 mo and was at a negligible prevalence of 2.8% at 18 mo, although estimates adjusted for protein quality and infection were more than twice as high (e.g. 25.1% at 9 mo and 7.0% at 18 mo). The proportion of children with inadequate intakes of protein, both unadjusted and adjusted for protein quality, was the highest in the control group compared with the supplemented groups at all timepoints. At 24 mo (post-supplementation), the protein inadequacy was somewhat higher than at 18 mo in the supplemented groups and lower than in the control group, albeit not significantly (Table 2). Across all groups and ages, lysine was the limiting amino acid in home-based complementary foods for >80% of children (Supplemental Figure 1). On the basis of the amino acid content of the CFSs, the DIAAS was 89.2, 88.6, and 83.9 for the CP, RL, and WSB++ products, respectively; sulfur amino acids were the limiting amino acid for CP and RL, and lysine for WSB++ (Supplemental Table 3).

The substitution of macronutrients from home-based complementary foods because of the CFS products was not observed – for most macronutrients, intakes from home foods were not different in the supplemented groups compared with the control group in energy-adjusted models (MANOVA *P* value >0.05) (Table 3) [13]. Energy intakes from home foods were somewhat higher in the CFS groups compared with the control, albeit not significantly.

**TABLE 3:** Estimated energy and select nutrient intakes from home complementary foods among children enrolled in a complementary food supplementation trial in Bangladesh, by age and supplementation group^1^.

	Mean (SD) energy and nutrient intakes					
	9 mo					
	CFC-only (*n* = 1215)	Plumpy'doz (*n* = 1301)	Chickpea (*n* = 715)	Rice-lentil (*n* = 727)	WSB++ (*n* = 727)	MANOVA *P value*
Energy (kcal)	230.0 (158.6)	247.7 (169.6)	258.6 (167.7)	253.9 (160.4)	251.6 (171.5)	0.001
Protein (g)	6.2 (5.1)	6.8 (5.7)	7.2 (5.5)	7.0 (5.2)	7.1 (5.9)	0.184
Fat (g)	6.2 (5.4)	6.9 (6.0)	7.0 (5.8)	6.9 (5.7)	7.0 (6.1)	0.608
Calcium (mg)	65.8 (77.3)	73.7 (88.3)	76.8 (82.5)	73.4 (81.3)	79.1 (91.0)	0.574
Magnesium (mg)	32.5 (24.7)	35.7 (27.5)	38.8 (29.6)	36.6 (26.4)	37.1 (28.7)	0.012
Phosphorus (mg)	103.9 (88.9)	114.9 (98.4)	119.4 (91.0)	116.9 (89.2)	120.9 (101.9)	0.263
Potassium (mg)	168.7 (149.9)	188.5 (171.9)	200.7 (179.8)	189.8 (159.7)	196.9 (175.8)	0.170

	12 mo					

	CFC-only (*n* = 1216)	Plumpy'doz (*n* = 1299)	Chickpea (*n* = 712)	Rice-lentil (*n* = 725)	WSB++ (*n* = 728)	MANOVA *P value*

Energy (kcal)	349.6 (218.3)	369.1 (219.5)	387.5 (223.0)	367.7 (209.1)	385.2 (222.9)	0.001
Protein (g)	9.3 (6.5)	9.9 (6.9)	10.3 (6.8)	9.9 (6.4)	10.2 (6.8)	0.889
Fat (g)	8.6 (6.8)	9.3 (7.2)	9.6 (7.1)	9.1 (6.6)	9.3 (6.9)	0.235
Calcium (mg)	102.8 (100.9)	105.4 (102.9)	114.5 (109.6)	107.8 (95.9)	109.7 (104.5)	0.533
Magnesium (mg)	54.9 (38.0)	57.7 (38.2)	61.5 (40.6)	58.1 (38.0)	61.6 (41.2)	0.526
Phosphorus (mg)	157.3 (113.7)	166.0 (115.4)	175.9 (118.2)	166.1 (108.0)	172.0 (116.4)	0.705
Potassium (mg)	293.4 (230.9)	315.3 (240.0)	319.5 (233.4)	305.5 (226.6)	318.3 (233.6)	0.504

	15 mo					

	CFC-only (*n* = 1219)	Plumpy'doz (*n* = 1299)	Chickpea (*n* = 711)	Rice-lentil (*n* = 725)	WSB++ (*n* = 730)	MANOVA *P value*

Energy (kcal)	446.3 (270.7)	478.0 (266.4)	480.6 (259.6)	477.6 (276.3)	507.8 (280.2)	0.000
Protein (g)	12.0 (8.1)	12.9 (8.2)	12.9 (7.9)	12.9 (8.5)	13.4 (8.2)	0.438
Fat (g)	11.2 (8.5)	11.9 (8.7)	11.9 (8.4)	11.8 (8.8)	12.1 (8.6)	0.117
Calcium (mg)	129.5 (120.1)	142.9 (133.9)	147.0 (131.1)	143.4 (130.1)	145.7 (117.5)	0.182
Magnesium (mg)	71.3 (49.8)	75.7 (46.5)	76.6 (47.7)	76.5 (50.4)	81.9 (52.5)	0.659
Phosphorus (mg)	201.8 (141.6)	217.9 (143.7)	221.7 (140.5)	217.4 (147.1)	224.7 (133.2)	0.035
Potassium (mg)	398.3 (302.6)	418.4 (295.1)	419.9 (290.3)	416.7 (301.0)	435.1 (290.0)	0.709

	18 mo					

	CFC-only (*n* = 1218)	Plumpy'doz (*n* = 1299)	Chickpea (*n* = 711)	Rice-lentil (*n* = 726)	WSB++ (*n* = 727)	MANOVA *P value*

Energy (kcal)	532.5 (289.6)	578.2 (293.9)	579.2 (315.8)	567.9 (300.5)	589.1 (305.0)	0.001
Protein (g)	14.4 (8.7)	15.9 (9.1)	15.4 (9.1)	15.4 (9.0)	16.0 (9.2)	0.106
Fat (g)	13.7 (9.6)	15.0 (9.9)	14.0 (10.0)	14.4 (9.4)	14.7 (9.7)	0.003
Calcium (mg)	151.1 (126.3)	166.5 (138.4)	160.7 (137.6)	164.7 (135.7)	171.0 (137.7)	0.551
Magnesium (mg)	80.2 (50.6)	87.0 (53.0)	89.3 (54.2)	88.1 (55.8)	89.2 (51.8)	0.129
Phosphorus (mg)	237.7 (142.3)	261.5 (152.3)	256.2 (154.2)	253.1 (146.5)	266.4 (154.2)	0.321
Potassium (mg)	430.0 (298.6)	460.3 (298.0)	459.8 (304.9)	455.8 (292.9)	467.7 (296.5)	0.881

Abbreviations: CFC, child feeding counseling; MANOVA, multivariate analysis of variance models; WSB++, wheat-soy blend.

1 Based on intakes from maternal-reported home complementary foods only, not including complementary food supplements or breastmilk. Macro- and micronutrients were adjusted for energy intake by regressing each nutrient on energy intake (in kcal) and then using the residuals in the MANOVA. *P* values are from MANOVA models with all nutrients included as dependent variables and allocation group as the independent variable, generated separately for each age. The model included 12 additional micronutrients, which are presented elsewhere [13].

At 9 mo of age, 76.0% of children in the unsupplemented group and ∼50% children in each of the supplemented groups did not meet the recommendation for fat intake (Supplemental Table 4). However, total estimated fat intake in the PD group was higher than the AI of 30 g/d, implying a low prevalence of intake inadequacy; estimated fat intakes in the CP and RL groups were also relatively close to the AI (Supplemental Table 2) [35]. The proportion not meeting the recommendation for carbohydrate intake decreased across groups as children aged and remained >30% at all ages (Supplemental Table 4).

Sensitivity analyses applying a range of breastmilk intake values showed that the proportion not meeting energy or protein requirements remained high at enrollment, even when assuming actual breastmilk intake was 20% greater than the published values for age-specific average breastmilk intake (i.e. 37.6%–46.3%, energy; 63.8%–66.2%, unadjusted protein; 78.0%–80.9%, adjusted for protein quality and infection); overall, the proportion not meeting requirements was significantly higher across ages in the unsupplemented group compared with the supplemented groups (Supplemental Tables 5–8).

## Discussion

In this rural setting in Bangladesh, energy and macronutrient intakes from home-based complementary foods increased and intake inadequacy decreased as children aged from 6 to 24 mo. Overall, there was a notable drop in protein inadequacy after 6 mo and the supplements virtually eliminated any intake gaps. Despite these positive trends, and even while receiving medium-quantity CFSs, ∼10%–12% of supplemented children experienced energy inadequacy at 18 mo of age and a third had inadequate energy intake by 24 mo, which was 6 mo post-supplementation. The provision of CFSs enabled more children to reach protein and energy intake requirements during the 1-y intervention period and did not result in substitution of home foods. These findings show the beneficial impact of CFSs in filling energy and macronutrient intake gaps in a context with dietary inadequacy from the home diet during the first 2 y of life.

The relatively high prevalence of energy inadequacy among children in the unsupplemented control group (74.6% at 6 mo, decreasing to 31.4% at 18 mo) reflects our previous findings that the home diet primarily consists of low energy density grains and starchy tubers, such as rice and potato [27]. The CFSs provided were designed to meet 20%–30% of estimated energy needs and to be consumed in addition to home foods and breastmilk; as expected, the proportion of children meeting energy requirements was higher among those receiving CFSs [12]. The CFSs doubled in portion size from 125 to 250 kcal/d starting at 12 mo of age, which seemed warranted, given that 6.0%–8.2% and 10.5%–13.8% of children in the supplemented groups still had inadequate energy intake at 15 and 18 mo, respectively. Concerns have been raised that increasing the energy density of the diet through CFSs may reduce breastmilk intake [36]. We did not observe a difference in breastfeeding frequency across groups in the trial [27]. As we did not measure the quantity of breastmilk consumption, we could not assess if the amount consumed was similar across arms and actual consumption may have differed from the average breastmilk intake values used. However, other trials providing CFSs that measured breastmilk intake did not find a difference in amount consumed by arm, concluding that CFSs do not affect the quantity of breastmilk consumed [[37], [38], [39]]. Similar conclusions can be made regarding the impact of the CFSs on protein intake, with almost all children in the supplemented groups meeting age-based requirements and fewer than ∼2.5% of the older children experiencing inadequacy even after adjusting for infection and protein quality.

Early research evaluating protein intake adequacy had led to conclusions that the amount of protein consumed during the complementary feeding period in LMICs is greater than requirements, assuming average breastmilk intake [4]. However, previous studies did not account for protein quality or infection, thereby likely overestimating adequacy. A re-analysis of dietary intake data collected from 6 LMICs adjusting for protein quality concluded that children consumed greater amounts of protein than required, except for breastfed 6–8-mo olds who consumed low amounts of complementary foods [40]. However, the study employed the Protein Digestibility-Corrected Amino Acid Score method, which is no longer recommended (although measures were taken to address this limitation), and did not evaluate the potential effects of acute infections or chronic conditions, which increase nutrient needs [31,40]. A modeling exercise using data from FAO Food Balance Sheets to estimate the influence of protein quality and infection on protein intake adequacy resulted in an increased prevalence of inadequacy, suggesting an under-recognized public health problem [34]. Our finding that protein intake inadequacy increased at each timepoint after adjusting for DIAAS and infection, most notably in the unsupplemented control group, indicates that protein may be a nutrient of concern in populations with poor diet quality and a high burden of infection. The finding that lysine was the amino acid with the highest prevalence of inadequacy for this population, and was the limiting amino acid for WSB++, was expected given lysine content is low in staples like rice and wheat.

The high prevalence of protein inadequacy (adjusted for protein quality and infection) at 6 mo of age at above 85% was an interesting finding, accompanied by a dramatic decrease at 9 mo to 25% in the unsupplemented control group likely largely linked to increased consumption of home-based complementary foods. Although our estimates were based on 1 d of intake at each visit, which did not allow for the estimate and removal of within-subject variation that would have increased precision, our findings are reflective of the changes in diet during the early complementary feeding period. For example, the proportion of children consuming fish, eggs, and milk in the last 24 h at 9 mo of age was 30.0%, 22.3%, and 13.6*%*, respectively, compared with 7.4%, 7.5%, and 8.7%, respectively, at 6 mo of age. Our findings also relate to the question of whether the current WHO recommendation of exclusive breastfeeding for the first 6 mo of life results in the protein needs of infants during 5–6 mo of age being unmet at 6 mo, as most children in our study were exclusively breastfed [27]. A 2012 meta-analysis informing the current WHO recommendation highlighted limitations in the overall evidence for the benefit of exclusive breastfeeding for 6 compared with 4–6 mo, which largely comprised a small number of studies that have predominantly been observational with the exception of 1 trial from Honduras, which showed no difference in weight or length indicating equivalency of the 4–6 mo compared with 6-mo duration rather than superiority [41]. Recent analyses from longitudinally followed birth cohorts show significant evidence of growth faltering and onset of stunting in the first 6 mo of life [42]. The significant protein gap observed in our study at 6 mo, along with the recognized need for further research, suggests a need to re-evaluate if a shift to a 4–6-mo exclusive breastfeeding recommendation is necessary. Additionally, focus should be placed on how to better incorporate foods rich in quality protein in the diet, considering their availability, accessibility, and acceptability.

The optimal intake of fat from birth to 2 y of age has not been defined, and there is currently no fat intake recommendation for 12–18 mo of age [2]. It is assumed that little fat is needed from complementary foods in early infancy because intake of breastmilk, rich in fat, is typically high. However, as breastmilk intake decreases with age, the quantity and quality of fat from complementary foods becomes more important. The poor fat quality of cereal-based diets common in LMICs may contribute to undernutrition [2]. Data from the Gambia, for example, showed complementary foods to contain low amounts of fat, with essential fatty acids of particular concern, resulting in a lack of fat and essential fatty acids in the diet as children transition from breastmilk to complementary foods [43]. LNS have been designed to be good sources of essential fatty acids, with focus on including alpha-linolenic acid as it has been found to have an important role in linear growth and cognitive development [8,44].

Overall, we did not observe reduced intake of home-based complementary foods, as reflected by energy and macronutrient intakes, because of supplementation with complementary foods in either a lipid-based or cooked fortified blended flour form in the study. However, the daily portions were small, providing an additional 125 kcal/d at least during the 6–12-mo period. Doubling that amount to 250 kcal/d from 12–18 mo did not change home-based food and macronutrient intake, suggesting that these quantities of CFSs did not result in substitution. These findings are consistent with results from our trial that also found no group-wise differences in food group or micronutrient intakes, as well as other studies on small-quantity LNS showing no substitution, further strengthening evidence that CFSs do not replace home foods [13,27,45,46]. Energy intakes from home-based complementary foods were slightly higher in the supplemented groups relative to the control, indicating a greater quantity of home foods consumed among children receiving the CFSs, which may have been because of increased appetite or increased body weight leading to higher energy needs.

There were some limitations to our study. We did not directly measure breastmilk intake and instead used age-specific average intake estimates with typical energy and macronutrient contents, which may have under- or overestimated inadequacies if actual intake differed from average values. However, our findings did not drastically differ after conducting sensitivity analyses using a range of breastmilk intake values. The 24-h diet questionnaire was not open-ended and may not have captured all foods consumed, potentially resulting in an underestimation of intakes. However, the method included the most commonly consumed foods, based on previous 7-d food-frequency data; allowed for additional foods to be captured; had been validated; and has been used to report on nutrient intakes and adequacies in other studies [13,47]. We also used data on local recipes and portion sizes to inform the conversion of food to nutrient intakes, and employed interviewers who were highly trained in collecting quantitative dietary data.

We collected 1 d of dietary intake data at each visit, so it was not possible to estimate within-subject variation, which would have allowed us to estimate usual intake distributions and increase precision in prevalence estimates. Data on intra- and inter-individual variability of nutrient intakes in children is limited, especially in LMICs. Estimates of variability are most useful when measured in the study population as they can vary by numerous factors, and data are needed on variability during the complementary feeding period when the diet is changing with the intermittent introduction of small amounts of new foods as children age. Studies that have measured components of variance in children’s intakes have reported low intra- to inter-individual variance ratios, indicating a higher contribution of inter-individual variability to total variance, and that the variance ratio is lower among younger compared with older children for most nutrients, implying the need for more dietary replications is greater among older children [[48], [49], [50], [51]]. By repeating measurements every 3 mo for over a year, our study was able to capture the changes that occur in dietary intakes as the types and amounts of foods consumed during the complementary feeding period change with age and development. Finally, the trial was unblinded, which may result in potential bias. However, we had a large sample size and longitudinal data, which are significant strengths. We used an estimation of number of infections, and not actual reported values, which could have influenced the precision of adjusted protein intakes.

Our findings on energy and macronutrient intakes mirror our findings on micronutrient intakes, thereby presenting a comprehensive characterization of dietary intakes and inadequacies in this population [13]. The impact of CFSs in filling energy and protein gaps that we observed shows how CFSs designed to deliver a combination of energy, high-quality protein and fat, and micronutrients can successfully address multiple nutritional deficiencies and their health implications. The main trial also found that CFSs had a beneficial impact on linear growth and nutritional status. Meta-analyses of trials providing LNS, which include ours, have concluded a reduced risk of multiple adverse health outcomes in early life, including mortality, severe wasting, severe stunting, iron deficiency anemia, and developmental delay [9,52,53]. Our study further adds to this evidence supporting the scale-up and integration of small- and medium-quantity LNS in preventive nutrition interventions for improved child health and well-being.

## Author contributions

The authors’ responsibilities were as follows – PC: designed the research; AAS, SS: were involved in implementation of the research; RKC: analyzed the data; MMP: wrote the paper; PC: had primary responsibility for final content; and all authors read and approved the final manuscript.

## Funding

This work was supported by the United States Department of Agriculture, National Institute of Food and Agriculture, under the Food and Nutrition Enhancement Program grant 2010-38418-21732. In-kind support was received from DSM (Basel, Switzerland) and Nutriset (Maulany, France). Additional support for the study was provided Johns Hopkins Sight and Life Global Nutrition Research Institute and Johns Hopkins Center for Global Health.

## Conflict of interest

The authors report no conflicts of interest.

## References

[1] Victora C.G., de Onis M., Hallal P.C., Blössner M., Shrimpton R. (2010). Worldwide timing of growth faltering: revisiting implications for interventions. Pediatrics.

[2] Dewey K.G., Adu-Afarwuah S. (2008). Systematic review of the efficacy and effectiveness of complementary feeding interventions in developing countries. Matern. Child. Nutr..

[3] Allen L.H. (2012). Adequacy of family foods for complementary feeding. Am. J. Clin. Nutr..

[4] Dewey K.G., Brown K.H. (2003). Update on technical issues concerning complementary feeding of young children in developing countries and implications for intervention programs. Food Nutr. Bull..

[5] Black R.E., Victora C.G., Walker S.P., Bhutta Z.A., Christian P., de Onis M. (2013). Maternal and child undernutrition and overweight in low-income and middle-income countries. Lancet.

[6] Keats E.C., Das J.K., Salam R.A., Lassi Z.S., Imdad A., Black R.E. (2021). Effective interventions to address maternal and child malnutrition: an update of the evidence, Lancet Child Adolesc. Health.

[7] Chaparro C.M., Dewey K.G. (2010). Use of lipid-based nutrient supplements (LNS) to improve the nutrient adequacy of general food distribution rations for vulnerable sub-groups in emergency settings. Matern. Child. Nutr..

[8] Arimond M., Zeilani M., Jungjohann S., Brown K.H., Ashorn P., Allen L.H. (2015). Considerations in developing lipid-based nutrient supplements for prevention of undernutrition: experience from the International Lipid-Based Nutrient Supplements (iLiNS) Project, Matern. Child. Nutr..

[9] Stewart C.P., Wessells K.R., Arnold C.D., Huybregts L., Ashorn P., Becquey E. (2020). Lipid-based nutrient supplements and all-cause mortality in children 6–24 months of age: a meta-analysis of randomized controlled trials. Am. J. Clin. Nutr..

[10] Dewey K.G., Stewart C.P., Wessells K.R., Prado E.L., Arnold C.D. (2021). Small-quantity lipid-based nutrient supplements for the prevention of child malnutrition and promotion of healthy development: overview of individual participant data meta-analysis and programmatic implications. Am. J. Clin. Nutr..

[11] Adams K.P., Vosti S.A., Arnold C.D., Engle-Stone R., Prado E.L., Stewart C.P. (2023). The cost-effectiveness of small-quantity lipid-based nutrient supplements for prevention of child death and malnutrition and promotion of healthy development: modelling results for Uganda. Public Health Nutr.

[12] Christian P., Shaikh S., Shamim A.A., Mehra S., Wu L., Mitra M. (2015). Effect of fortified complementary food supplementation on child growth in rural Bangladesh: a cluster-randomized trial. Int. J. Epidemiol..

[13] Campbell R.K., Hurley K.M., Shamim A.A., Shaikh S., Chowdhury Z.T., Mehra S. (2018). Complementary food supplements affect dietary nutrient adequacy and home food consumption in children 6–18 mo old in a randomized controlled trial in rural Bangladesh. J. Nutr..

[14] Gibson R.S., Charrondiere U.R., Bell W. (2017). Measurement errors in dietary assessment using self-reported 24-hour recalls in low-income countries and strategies for their prevention. Adv. Nutr..

[15] Labrique A.B., Christian P., Klemm R.D., Rashid M., Shamim A.A., Massie A. (2011). A cluster-randomized, placebo-controlled, maternal vitamin A or beta-carotene supplementation trial in Bangladesh: design and methods. Trials.

[16] National Institute of Population Research and Training - NIPORT/Bangladesh (2020).

[17] Merrill R.D., Shamim A.A., Ali H., Jahan N., Labrique A.B., Schulze K. (2011). Iron status of women is associated with the iron concentration of potable groundwater in rural Bangladesh. J. Nutr..

[18] Menon P., Nguyen P.H., Saha K.K., Khaled A., Sanghvi T., Baker J. (2016). Combining intensive counseling by frontline workers with a nationwide mass media campaign has large differential impacts on complementary feeding practices but not on child growth: results of a cluster-randomized program evaluation in Bangladesh. J. Nutr..

[19] Chowdhury Z.T., Hurley K.M., Campbell R.K., Shaikh S., Shamim A.A., Mehra S. (2020). Novel method for estimating nutrient intakes using a semistructured 24-hour diet recall for infants and young children in rural Bangladesh. Curr. Dev. Nutr..

[20] Shaheen N., Rahim A., Mohiduzzaman M., Banu C., Bari M., Tukun A. (2013).

[21] Gopalan C., Rama Sastri B., Balasubramanian S. (1971).

[22] Public Health England (2002).

[23] Institue of Nutrition Mahidol University (2014).

[24] Lukmanji Z., Herzmark E., Mlingi N., Assey V., Ndossi G., Fawzi W. (2008).

[25] National Nutrition Program (2012).

[26] US Department of Agriculture Agricultural Research Service (2013).

[27] Campbell R.K., Hurley K.M., Shamim A.A., Shaikh S., Chowdhury Z.T., Mehra S. (2016). Effect of complementary food supplementation on breastfeeding and home diet in rural Bangladeshi children. Am. J. Clin. Nutr..

[28] WHO (1998).

[29] FAO. Human Energy Requirements (2004).

[30] Institute of Medicine (2006).

[31] FAO (2013).

[32] FAO. Protein Quality Evaluation (1991).

[33] Millward D.J., Jackson A.A. (2004). Protein/energy ratios of current diets in developed and developing countries compared with a safe protein/energy ratio: implications for recommended protein and amino acid intakes. Public Health Nutr.

[34] Ghosh S., Suri D., Uauy R. (2012). Assessment of protein adequacy in developing countries: quality matters. Br. J. Nutr..

[35] Murphy S.P., Guenther P.M., Kretsch M.J. (2006). Using the dietary reference intakes to assess intakes of groups: pitfalls to avoid. J. Am. Diet. Assoc..

[36] Islam M.M., Khatun M., Peerson J.M., Ahmed T., Mollah M.A., Dewey K.G. (2008). Effects of energy density and feeding frequency of complementary foods on total daily energy intakes and consumption of breast milk by healthy breastfed Bangladeshi children. Am. J. Clin. Nutr..

[37] Kumwenda C., Dewey K.G., Hemsworth J., Ashorn P., Maleta K., Haskell M.J. (2014). Lipid-based nutrient supplements do not decrease breast milk intake of Malawian infants. Am. J. Clin. Nutr..

[38] Galpin L., Thakwalakwa C., Phuka J., Ashorn P., Maleta K., Wong W.W., Manary M.J. (2007). Breast milk intake is not reduced more by the introduction of energy dense complementary food than by typical infant porridge. J. Nutr..

[39] Owino V.O., Bahwere P., Bisimwa G., Mwangi C.M., Collins S. (2011). Breast-milk intake of 9–10-mo-old rural infants given a ready-to-use complementary food in South Kivu, Democratic Republic of Congo. Am. J. Clin. Nutr..

[40] Arsenault J.E., Brown K.H. (2017). Dietary protein intake in young children in selected low-income countries is generally adequate in relation to estimated requirements for healthy children, except when complementary food intake is low. J. Nutr..

[41] Kramer M.S., Kakuma R. (2012). Optimal duration of exclusive breastfeeding. Cochrane Database Syst. Rev..

[42] Mertens A., Benjamin-Chung J., Colford J.M., Coyle J., van der Laan M.J., Hubbard A.E. (2023). Causes and consequences of child growth faltering in low-resource settings. Nature.

[43] Prentice A.M., Paul A.A. (2000). Fat and energy needs of children in developing countries. Am. J. Clin. Nutr..

[44] Adu-Afarwuah S., Lartey A., Brown K.H., Zlotkin S., Briend A., Dewey K.G. (2007). A fat-based supplement containing essential fatty acids increased plasma α-linolenic acid and linear growth of Ghanaian infants. FASEB J.

[45] Flax V.L., Siega-Riz A.M., Reinhart G.A., Bentley M.E. (2015). Provision of lipid-based nutrient supplements to Honduran children increases their dietary macro- and micronutrient intake without displacing other foods. Matern. Child. Nutr..

[46] Ickes S.B., Adair L.S., Brahe C.A., Thirumurthy H., Charles B., Myhre J.A. (2015). Impact of lipid-based nutrient supplementation (LNS) on children's diet adequacy in Western Uganda, Matern. Child. Nutr..

[47] Pasqualino M.M., Shaikh S., McGready J., Islam M.T., Ali H., Ahmed T. (2023). An egg intervention improves dietary intakes but does not fill intake gaps for multiple micronutrients among infants in rural Bangladesh. J. Nutr..

[48] Padilha L.L., França A.K., da Conceição S.I., Carvalho W.R., Batalha M.A., da Silva A.A. (2017). Nutrient intake variability and the number of days needed to estimate usual intake in children aged 13–32 months. Br. J. Nutr..

[49] Huybrechts I., De Bacquer D., Cox B., Temme E.H., Van Oyen H., De Backer G. (2008). Variation in energy and nutrient intakes among pre-school children: implications for study design. Eur. J. Public Health..

[50] Nelson M., Black A.E., Morris J.A., Cole T.J. (1989). Between- and within-subject variation in nutrient intake from infancy to old age: estimating the number of days required to rank dietary intakes with desired precision. Am. J. Clin. Nutr..

[51] Jahns L., Carriquiry A., Arab L., Mroz T.A., Popkin B.M. (2004). Within- and between-person variation in nutrient intakes of Russian and U.S. children differs by sex and age. J. Nutr..

[52] Dewey K.G., Arnold C.D., Wessells K.R., Prado E.L., Abbeddou S., Adu-Afarwuah S. (2022). Preventive small-quantity lipid-based nutrient supplements reduce severe wasting and severe stunting among young children: an individual participant data meta-analysis of randomized controlled trials. Am. J. Clin. Nutr..

[53] Das J.K., Salam R.A., Hadi Y.B., Sadiq Sheikh S., Bhutta A.Z., Weise Prinzo Z. (2019). Preventive lipid-based nutrient supplements given with complementary foods to infants and young children 6 to 23 months of age for health, nutrition, and developmental outcomes. Cochrane Database Syst. Rev..

