# Supplementary material for: Complementary Food Supplements Fill Energy and Protein Gaps among Children with Dietary Inadequacy in a Complementary Feeding Trial in Rural Bangladesh
Source: J Nutr. 2024 Dec 9;155(2):602–11. doi: 10.1016/j.tjnut.2024.12.001 (PMC13168993; doi:10.1016/j.tjnut.2024.12.001)
Supplement: multimedia component 1 [file mmc1.docx]

**
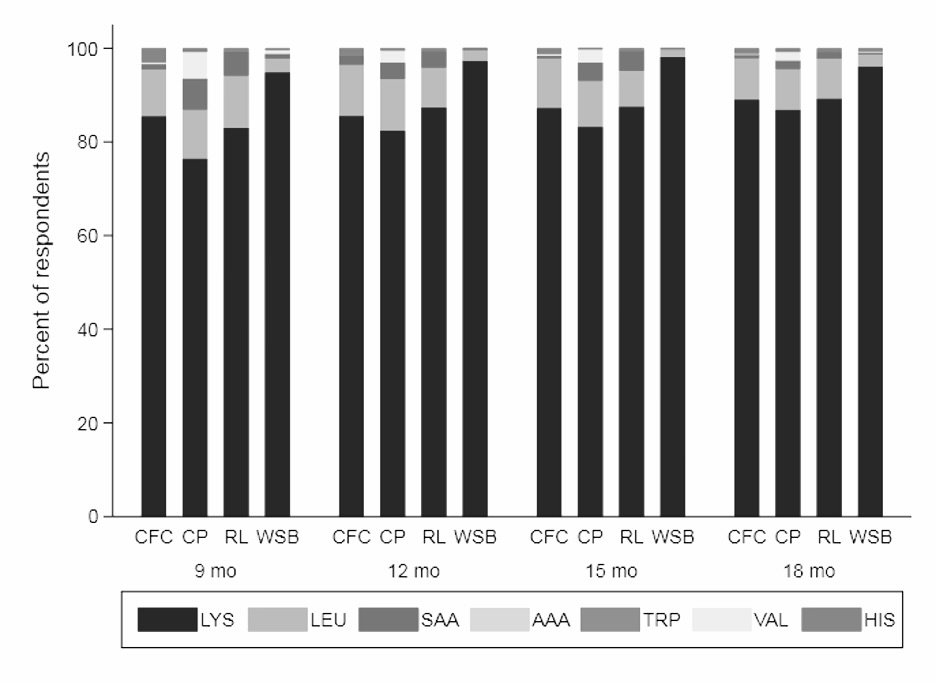
**

**Supplemental Figure 1.** Limiting amino acid in complementary foods by supplementation group and age.^1^

^1^Complementary food intakes included home foods and complementary food supplements (CFSs) in the supplemented groups. CFS intakes were calculated using maternal-reported percentage of daily portion consumed for the day of the 24-hour diet questionnaire. Children with zero reported protein intake in the diet questionnaire were omitted from the figure. Percentages represent the amino acid in lowest supply relative to the reference profile in reported protein intake from home foods and CFSs.

| **Supplemental Table 1.** Baseline parental and household characteristics of enrolled children by CFS group (n=5,421) | | | | | | | | | | |
| --- | --- | --- | --- | --- | --- | --- | --- | --- | --- | --- |
|  | Control | | Plumpy’doz | | Rice lentil | | Chickpea | | WSB++ | |
| Maximum n | 1428 | | 1484 | | 817 | | 837 | | 839 | |
|  | Mean | SD | Mean | SD | Mean | SD | Mean | SD | Mean | SD |
| Household size | 5.0 | 1.7 | 5.0 | 1.8 | 5.0 | 1.8 | 5.1 | 1.9 | 5.0 | 1.8 |
| Paternal age | 30.9 | 7.1 | 31.2 | 7.5 | 30.8 | 7.1 | 31.2 | 7.1 | 30.9 | 7.1 |
| Maternal age | 24.3 | 5.6 | 24.1 | 5.4 | 24.2 | 5.4 | 24.1 | 5.3 | 24.1 | 5.4 |
|  | *n* | % | *n* | % | *n* | % | *n* | % | *n* | % |
| Maternal education | | | | | | | | | | |
| No schooling | 357 | 25.1 | 361 | 24.3 | 190 | 23.3 | 193 | 23.1 | 197 | 23.6 |
| Class 1 to 9 | 916 | 64.3 | 950 | 64.0 | 546 | 66.9 | 543 | 64.9 | 533 | 63.8 |
| SSC Passed | 77 | 5.4 | 74 | 5.0 | 31 | 3.8 | 47 | 5.6 | 41 | 4.9 |
| 11 years and above | 75 | 5.3 | 99 | 6.7 | 49 | 6.0 | 54 | 6.4 | 65 | 7.8 |
| Religion |  |  |  |  |  |  |  |  |  |  |
| Muslim | 1319 | 92.4 | 1374 | 92.6 | 762 | 93.3 | 754 | 90.1 | 778 | 92.7 |
| Hindu | 107 | 7.5 | 108 | 7.3 | 51 | 6.2 | 83 | 9.9 | 60 | 7.2 |
| Other | 2 | 0.1 | 2 | 0.1 | 4 | 0.5 | 0 | 0 | 1 | 0.1 |
| Household food insecurity^1^ | | | | | | | | | | |
| HFI 9 | 712 | 49.9 | 760 | 51.2 | 429 | 52.5 | 432 | 51.6 | 418 | 49.8 |
| HFI 10-15 | 520 | 36.4 | 537 | 36.2 | 291 | 35.6 | 294 | 35.1 | 309 | 36.8 |
| HFI ≥16 | 196 | 13.7 | 187 | 12.6 | 97 | 11.9 | 111 | 13.3 | 112 | 13.4 |
| Assets |  |  |  |  |  |  |  |  |  |  |
| Cattle^2^ | 701 | 49.1 | 774 | 52.2 | 408 | 49.9 | 418 | 49.9 | 452 | 53.9 |
| Any land | 996 | 69.8 | 1046 | 70.6 | 563 | 69.0 | 608 | 72.7 | 566 | 67.5 |
| Electricity | 450 | 31.5 | 480 | 32.4 | 252 | 30.9 | 272 | 32.5 | 233 | 27.8 |
| SSC, Secondary School Certificate.  ^1^HFI, Household Food Insecurity assessed using a modified questionnaire with nine questions by summing five ordered responses (1. Never, 2. Rarely, 3. Sometimes, 4. Often and 5. Mostly); HFI score ranged from 9 to its possible maximum of 45 (50).  ^2^Using GEE linear or multinomial logistic regression analysis, goat/sheep ownership *P*-value: 0.009. | | | | | | | | | | |

| **Supplemental Table 2.** Estimated total energy and macronutrient intakes from breastmilk, home complementary foods, and complementary food supplements (in supplemented groups) among children enrolled in a complementary food supplementation trial in Bangladesh, by age and supplementation group^1^ | | | | | | | |
| --- | --- | --- | --- | --- | --- | --- | --- |
|  | 9 mo |  | 12 mo |  | 15 mo |  | 18 mo |
|  | Median (25th, 75th pctl) |  | Median (25th, 75th pctl) |  | Median (25th, 75th pctl) |  | Median (25th, 75th pctl) |
| Group | Energy (kcal/kg/d) | | | | | | |
| CFC-only | 79.5 (66.2 , 97.1) |  | 84.8 (68.3 , 106.0) |  | 91.9 (72.2 , 116.4) |  | 98.8 (76.8 , 122.4) |
| Plumpy'doz | 88.4 (74.4 , 107.1) |  | 111.2 (92.1 , 132.5) |  | 122.4 (102.2 , 147.5) |  | 120.5 (97.4 , 145.7) |
| Chickpea | 90.9 (75.6 , 109.4) |  | 113.9 (97.6 , 137.5) |  | 125.3 (104.3 , 148.9) |  | 120.9 (96.8 , 150.6) |
| Rice-lentil | 91.2 (76.8 , 106.7) |  | 111.0 (93.3 , 130.6) |  | 120.8 (101.4 , 148.5) |  | 120.6 (98.3 , 146.8) |
| WSB++ | 89.8 (75.8 , 109.1) |  | 114.4 (95.1 , 138.2) |  | 127.2 (107.3 , 155.9) |  | 125.9 (99.7 , 151.0) |
|  | Unadjusted protein (g/d) | | | | | | |
| CFC-only | 11.5 (8.8 , 15.1) |  | 13.7 (10.3 , 18.7) |  | 16.6 (11.9 , 22.4) |  | 18.9 (13.7 , 25.7) |
| Plumpy'doz | 13.3 (10.5 , 17.5) |  | 19.1 (15.3 , 24.5) |  | 23.0 (18.2 , 28.7) |  | 24.7 (18.8 , 31.4) |
| Chickpea | 14.1 (10.9 , 18.5) |  | 20.5 (16.7 , 25.4) |  | 24.3 (19.4 , 29.9) |  | 24.3 (18.7 , 31.5) |
| Rice-lentil | 13.6 (10.6 , 17.7) |  | 18.2 (14.8 , 23.3) |  | 21.6 (17.2 , 28.4) |  | 23.7 (17.9 , 30.3) |
| WSB++ | 14.7 (11.8 , 20.0) |  | 23.9 (19.6 , 29.4) |  | 29.1 (23.8 , 35.4) |  | 29.3 (21.5 , 36.6) |
|  | DIAAS-adjusted protein (g/d)^2^ | | | | | | |
| CFC-only | 10.6 (8.2 , 14.3) |  | 12.6 (9.1 , 17.3) |  | 15.1 (10.6 , 20.2) |  | 17.0 (12.2 , 22.8) |
| Plumpy'doz | - |  | - |  | - |  | - |
| Chickpea | 13.1 (10.2 , 17.1) |  | 19.3 (15.6 , 24.2) |  | 22.8 (17.8 , 27.7) |  | 22.1 (17.2 , 29.3) |
| Rice-lentil | 12.3 (9.8 , 16.4) |  | 17.1 (13.7 , 22.1) |  | 20.4 (15.6 , 26.6) |  | 21.2 (16.0 , 27.9) |
| WSB++ | 13.4 (10.7 , 18.0) |  | 21.1 (17.0 , 26.5) |  | 25.0 (20.6 , 31.5) |  | 25.8 (18.9 , 32.1) |
|  | Fat (g/d) | | | | | | |
| CFC-only | 25.8 (23.1 , 29.7) |  | 26.1 (22.7 , 31.2) |  | 28.6 (24.1 , 34.9) |  | 31.4 (25.7 , 37.9) |
| Plumpy'doz | 30.2 (27.4 , 34.5) |  | 40.3 (35.5 , 45.6) |  | 44.6 (39.9 , 50.8) |  | 44.4 (37.1 , 52.3) |
| Chickpea | 29.7 (26.5 , 34.5) |  | 38.0 (33.7 , 43.3) |  | 41.8 (37.1 , 47.8) |  | 40.7 (33.9 , 48.3) |
| Rice-lentil | 29.3 (26.6 , 33.8) |  | 36.7 (32.2 , 42.0) |  | 40.6 (36.0 , 46.9) |  | 41.3 (34.2 , 48.3) |
| WSB++ | 27.6 (25.0 , 32.9) |  | 31.9 (27.9 , 36.9) |  | 35.5 (30.9 , 42.2) |  | 36.7 (31.0 , 43.6) |
|  | Carbohydrate (g/d) | | | | | | |
| CFC-only | 71.7 (57.1 , 93.4) |  | 88.8 (67.5 , 116.7) |  | 102.9 (77.4 , 135.7) |  | 116.7 (87.7 , 154.8) |
| Plumpy'doz | 79.4 (63.0 , 101.8) |  | 107.3 (86.6 , 136.3) |  | 127.4 (101.8 , 162.5) |  | 138.5 (108.4 , 176.4) |
| Chickpea | 84.4 (66.2 , 104.9) |  | 117.3 (95.2 , 146.2) |  | 136.2 (109.9 , 168.3) |  | 142.1 (111.8 , 193.5) |
| Rice-lentil | 82.1 (69.6 , 102.3) |  | 114.7 (92.4 , 137.9) |  | 132.4 (109.6 , 169.6) |  | 142.0 (109.3 , 181.3) |
| WSB++ | 84.1 (68.1 , 105.8) |  | 126.5 (102.3 , 155.4) |  | 150.5 (120.2 , 189.0) |  | 151.8 (119.1 , 195.9) |
| ^1^Based on estimated total macronutrient intake, which included maternal-reported home food intake, maternal-reported complementary food supplement quantity consumed, and average breastmilk intake values from WHO, and intake recommendations from the Institute of Medicine. ^2^Estimated using the Digestible Indispensable Amino Acid Score method. Information not available to estimate available protein for Plumpy'doz. | | | | | | | |

| **Supplemental Table 3**. Amino acid contents and DIAAS of complementary food supplements**^1^** | | | |
| --- | --- | --- | --- |
|  | Complementary Food Supplement | | |
|  | Chickpea | Rice-lentil | WSB++ |
| Isoleucine (mg/g) | 1.242 | 1.275 | 1.256 |
| Leucine (mg/g) | 0.994 | 1.045 | 1.057 |
| Lysine (mg/g) | 1.046 | 1.022 | 0.839 |
| Sulphur amino acids  (methionine + cysteine, mg/g) | 0.892 | 0.886 | 1.088 |
| Aromatic amino acids (phenylalanine + tyrosine, mg/g) | 1.355 | 1.369 | 1.469 |
| Threonine (mg/g) | 1.073 | 1.058 | 1.087 |
| Tryptophan (mg/g) | 1.056 | 1.071 | 1.443 |
| Valine (mg/g) | 0.933 | 1.111 | 1.004 |
| Histidine (mg/g) | 1.183 | 1.182 | 1.097 |
| DIAAS | 89.2 (SAA) | 88.6 (SAA) | 83.9 (LYS) |
| DIAAS, Digestible Indispensable Amino Acid Score; WSB++, Wheat-Soy Blend; SAA, sulphur amino acids; LYS, lysine ^1^Information is not available to estimate DIAAS for Plumpy'doz. | | | |

| **Supplemental Table 4.** Proportion not meeting carbohydrate intake recommendations by age and supplement group among children enrolled in a complementary food supplementation trial in Bangladesh^1^ | | | | | | | |  |
| --- | --- | --- | --- | --- | --- | --- | --- | --- |
|  | 9 mo |  | 12 mo |  | 15 mo |  | 18 mo | |
|  | Fat^2^ | | | | | | | |
| Group | <AI (30 g/d)  n (%) |  |  |  |  |  |  | |
| CFC-only | 923 (76.0) |  | - |  | - |  | - | |
| Plumpy'doz | 634 (48.7) |  | - |  | - |  | - | |
| Chickpea | 377 (52.7) |  | - |  | - |  | - | |
| Rice-lentil | 406 (55.8) |  | - |  | - |  | - | |
|  | Carbohydrate | | | | | | | |
| Group | <AI (95 g/d)  n (%) |  | <RDA (130 g/d)  n (%) | | | | | |
| CFC-only | 934 (76.9) |  | 1013 (83.3) |  | 877 (71.9) |  | 725 (59.5) | |
| Plumpy'doz | 893 (68.6) |  | 925 (71.2) |  | 672 (51.7) |  | 574 (44.2) | |
| Chickpea | 450 (62.9) |  | 451 (63.3) |  | 311 (43.7) |  | 294 (41.4) | |
| Rice-lentil | 475 (65.3) |  | 504 (69.5) |  | 351 (48.4) |  | 299 (41.2) | |
| WSB++ | 450 (61.9) |  | 397 (54.5) |  | 245 (33.6) |  | 232 (31.9) | |
| AI, Adequate Intake; RDA, Recommended Dietary Allowance.  **^1^**Based on estimated total macronutrient intake, which included maternal-reported home food intake, maternal-reported complementary food supplement quantity consumed, and average breastmilk intake values from WHO, and intake recommendations from the Institute of Medicine (30, 32).  ^2^No intake recommendation has been set by the Institute of Medicine for 12 to 18 months of age (32). | | | | | | | |  |

| **Supplemental Table 5**. Prevalence of inadequate energy intakes by age and supplement group among children enrolled in a complementary food supplementation trial in Bangladesh^1^ | | | | | | | | | | | | | | |  |
| --- | --- | --- | --- | --- | --- | --- | --- | --- | --- | --- | --- | --- | --- | --- | --- |
|  |  | Assumed breastmilk intake as a % of the WHO 1998 average intake level | | | | | | | | | | | | | |
| Age | EAR, g/d^2^ | 100% | | 80% | | 90% | | 95% | | 105% | | 110% | | 120% | |
|  |  | Inadequate intake, % (n) | Chi^2^ p-value^3^ | Inadequate intake, % (n) | Chi^2^ p-value^3^ | Inadequate intake, % (n) | Chi^2^ p-value^3^ | Inadequate intake, % (n) | Chi^2^ p-value^3^ | Inadequate intake, % (n) | Chi^2^ p-value^3^ | Inadequate intake, % (n) | Chi^2^ p-value^3^ | Inadequate intake, % (n) | Chi^2^ p-value^3^ |
| 6 mo | 520.5 |  | 0.0001 |  | 0.0007 |  | 0.0000 |  | 0.0000 |  | 0.0000 |  | 0.0000 |  | 0.0000 |
| CFC-only |  | 1035 (74.6) |  | 1228 (88.5) |  | 1137 (82.0) |  | 1093 (78.8) |  | 961 (69.3) |  | 826 (59.6) |  | 521 (37.6) |  |
| Plumpy’doz |  | 1181 (81.3) |  | 1346 (92.7) |  | 1275 (87.8) |  | 1234 (85.0) |  | 1120 (77.1) |  | 1007 (69.4) |  | 685 (47.2) |  |
| Chickpea |  | 646 (78.7) |  | 736 (89.6) |  | 694 (84.5) |  | 670 (81.6) |  | 602 (73.3) |  | 542 (66.0) |  | 363 (44.2) |  |
| Rice-lentil |  | 647 (80.5) |  | 742 (92.3) |  | 707 (87.9) |  | 681 (84.7) |  | 613 (76.2) |  | 560 (69.7) |  | 368 (45.8) |  |
| WSB++ |  | 670 (80.6) |  | 762 (91.7) |  | 724 (87.1) |  | 706 (85.0) |  | 626 (75.3) |  | 581 (69.9) |  | 385 (46.3) |  |
| 9 mo | 573.5 |  | 0.0000 |  | 0.0000 |  | 0.0000 |  | 0.0000 |  | 0.0000 |  | 0.0000 |  | 0.0000 |
| CFC-only |  | 651 (48.7) |  | 881 (65.9) |  | 768 (57.4) |  | 707 (52.9) |  | 594 (44.4) |  | 511 (38.2) |  | 378 (28.3) |  |
| Plumpy’doz |  | 226 (16.2) |  | 485 (34.8) |  | 338 (24.2) |  | 280 (20.1) |  | 165 (11.8) |  | 122 (8.8) |  | 25 (1.8) |  |
| Chickpea |  | 115 (14.7) |  | 243 (31.2) |  | 165 (21.2) |  | 145 (18.6) |  | 83 (10.6) |  | 54 (6.9) |  | 11 (1.4) |  |
| Rice-lentil |  | 109 (14.0) |  | 245 (31.6) |  | 175 (22.6) |  | 134 (17.3) |  | 90 (11.6) |  | 73 (9.4) |  | 35 (4.5) |  |
| WSB++ |  | 125 (16.0) |  | 267 (34.2) |  | 187 (24.0) |  | 157 (20.1) |  | 89 (11.4) |  | 72 (9.2) |  | 22 (2.8) |  |
| 12 mo | 635.0 |  | 0.0000 |  | 0.0000 |  | 0.0000 |  | 0.0000 |  | 0.0000 |  | 0.0000 |  | 0.0000 |
| CFC-only |  | 602 (45.3) |  | 779 (58.7) |  | 697 (52.5) |  | 651 (49.0) |  | 554 (41.7) |  | 517 (38.9) |  | 433 (32.6) |  |
| Plumpy’doz |  | 163 (11.7) |  | 274 (19.7) |  | 214 (15.4) |  | 194 (13.9) |  | 127 (9.1) |  | 104 (7.5) |  | 74 (5.3) |  |
| Chickpea |  | 68 (8.6) |  | 134 (16.9) |  | 98 (12.3) |  | 81 (10.2) |  | 57 (7.2) |  | 45 (5.7) |  | 24 (3.0) |  |
| Rice-lentil |  | 100 (12.8) |  | 150 (19.3) |  | 122 (15.7) |  | 113 (14.5) |  | 84 (10.8) |  | 71 (9.1) |  | 39 (5.0) |  |
| WSB++ |  | 74 (9.4) |  | 143 (18.1) |  | 101 (12.8) |  | 82 (10.4) |  | 66 (8.3) |  | 50 (6.3) |  | 38 (4.8) |  |
| 15 mo | 665.0 |  | 0.0000 |  | 0.0000 |  | 0.0000 |  | 0.0000 |  | 0.0000 |  | 0.0000 |  | 0.0000 |
| CFC-only |  | 469 (35.9) |  | 643 (49.2) |  | 560 (42.8) |  | 509 (38.9) |  | 422 (32.3) |  | 388 (29.7) |  | 329 (25.2) |  |
| Plumpy’doz |  | 82 (6.0) |  | 148 (10.7) |  | 109 (7.9) |  | 98 (7.1) |  | 67 (4.9) |  | 55 (4.0) |  | 37 (2.7) |  |
| Chickpea |  | 46 (5.9) |  | 79 (10.2) |  | 63 (8.1) |  | 57 (7.3) |  | 41 (5.3) |  | 38 (4.9) |  | 23 (3.0) |  |
| Rice-lentil |  | 63 (8.2) |  | 107 (13.9) |  | 81 (10.5) |  | 70 (9.1) |  | 57 (7.4) |  | 50 (6.5) |  | 33 (4.3) |  |
| WSB++ |  | 47 (6.0) |  | 80 (10.2) |  | 63 (8.0) |  | 57 (7.2) |  | 37 (4.7) |  | 34 (4.3) |  | 20 (2.5) |  |
| 18 mo | 704.1 |  | 0.0000 |  | 0.0000 |  | 0.0000 |  | 0.0000 |  | 0.0000 |  | 0.0000 |  | 0.0000 |
| CFC-only |  | 399 (31.4) |  | 532 (41.9) |  | 471 (37.1) |  | 433 (34.1) |  | 367 (28.9) |  | 331 (26.0) |  | 267 (21.0) |  |
| Plumpy’doz |  | 140 (10.5) |  | 224 (16.8) |  | 171 (12.8) |  | 157 (11.8) |  | 121 (9.1) |  | 113 (8.5) |  | 84 (6.3) |  |
| Chickpea |  | 96 (12.7) |  | 136 (17.9) |  | 116 (15.3) |  | 99 (13.1) |  | 91 (12.0) |  | 80 (10.6) |  | 63 (8.3) |  |
| Rice-lentil |  | 104 (13.8) |  | 147 (19.4) |  | 121 (16.0) |  | 112 (14.8) |  | 91 (12.0) |  | 80 (10.6) |  | 57 (7.5) |  |
| WSB++ |  | 84 (11.1) |  | 128 (16.8) |  | 105 (13.8) |  | 90 (11.8) |  | 76 (10.0) |  | 70 (9.2) |  | 56 (7.4) |  |
| 24 mo | 778.0 |  | 0.0033 |  | 0.0056 |  | 0.0031 |  | 0.0037 |  | 0.0090 |  | 0.0252 |  | 0.0460 |
| CFC-only |  | 335 (27.1) |  | 441 (35.7) |  | 385 (31.1) |  | 358 (28.9) |  | 310 (25.1) |  | 276 (22.3) |  | 232 (18.8) |  |
| Plumpy’doz |  | 298 (23.9) |  | 404 (32.4) |  | 343 (27.6) |  | 322 (25.9) |  | 274 (22.0) |  | 259 (20.8) |  | 205 (16.5) |  |
| Chickpea |  | 175 (25.0) |  | 238 (34.0) |  | 206 (29.4) |  | 191 (27.3) |  | 161 (23.0) |  | 153 (21.9) |  | 128 (18.3) |  |
| Rice-lentil |  | 191 (26.9) |  | 264 (37.2) |  | 226 (31.8) |  | 211 (29.7) |  | 179 (25.2) |  | 170 (23.9) |  | 138 (19.4) |  |
| WSB++ |  | 145 (19.7) |  | 212 (28.8) |  | 176 (23.9) |  | 161 (21.9) |  | 137 (18.6) |  | 127 (17.3) |  | 105 (14.3) |  |

CFC, child feeding counseling; EAR, Estimated Average Requirement; WSB++, Wheat-Soy Blend.

^1^Energy and protein intakes include reported complementary food intake from a 24-hr recall, reported daily supplement intake, and age-specific estimated breast milk intake. From 6 to 11 months, the supplements were delivered in small quantities of 125 kcal/day and 2.6 g of protein. From 12 to 18 months, the supplements were delivered in medium quantities of 250 kcal/day and 5.2 g of protein. It was not possible to estimate adjusted protein intake for the group receiving PD from 9 to 18 months because information is not available to estimate the DIAAS for this commercially produced product.

^2^Estimated using mean weight of control group infants and children at each age time point.

^3^Chi^2^ p-values are from log binomial regression models with generalized estimating equations to allow for clustering of observations within sectors.

| **Supplemental Table 6**. Prevalence of inadequate protein intakes (unadjusted) by age and supplement group among children enrolled in a complementary food supplementation trial in Bangladesh^1^ | | | | | | | | | | | | | | | |
| --- | --- | --- | --- | --- | --- | --- | --- | --- | --- | --- | --- | --- | --- | --- | --- |
|  |  | Assumed breastmilk intake as a % of the WHO 1998 average intake level | | | | | | | | | | | | | |
| Age | EAR, g/d^2^ | 100% | | 80% | | 90% | | 95% | | 105% | | 110% | | 120% | |
|  |  | Inadequate intake, % (n) | Chi^2^ p-value^3^ | Inadequate intake, % (n) | Chi^2^ p-value^3^ | Inadequate intake, % (n) | Chi^2^ p-value^3^ | Inadequate intake, % (n) | Chi^2^ p-value^3^ | Inadequate intake, % (n) | Chi^2^ p-value^3^ | Inadequate intake, % (n) | Chi^2^ p-value^3^ | Inadequate intake, % (n) | Chi^2^ p-value^3^ |
| 6 mo | 10.3 |  | 0.9653 |  | 0.3593 |  | 0.6713 |  | 0.8668 |  | 0.8295 |  | 0.9126 |  | 0.8415 |
| CFC-only |  | 859 (78.4) |  | 947 (86.5) |  | 915 (83.6) |  | 891 (81.4) |  | 831 (75.9) |  | 793 (72.4) |  | 701 (64.0) |  |
| Plumpy’doz |  | 786 (78.1) |  | 864 (85.8) |  | 822 (81.6) |  | 806 (80.0) |  | 762 (75.7) |  | 729 (72.4) |  | 642 (63.8) |  |
| Chickpea |  | 477 (78.2) |  | 509 (83.4) |  | 496 (81.3) |  | 485 (79.5) |  | 457 (74.9) |  | 442 (72.5) |  | 404 (66.2) |  |
| Rice-lentil |  | 442 (79.2) |  | 479 (85.8) |  | 462 (82.8) |  | 453 (81.2) |  | 430 (77.1) |  | 409 (73.3) |  | 364 (65.2) |  |
| WSB++ |  | 453 (77.4) |  | 511 (87.4) |  | 488 (83.4) |  | 474 (81.0) |  | 434 (74.2) |  | 414 (70.8) |  | 373 (63.8) |  |
| 9 mo | 7.4 |  | 0.0000 |  | 0.0000 |  | 0.0000 |  | 0.0000 |  | 0.0000 |  | 0.0000 |  |  |
| CFC-only |  | 107 (8.3) |  | 283 (21.9) |  | 193 (14.9) |  | 154 (11.9) |  | 58 (4.5) |  | 25 (1.9) |  | 0 (0) |  |
| Plumpy’doz |  | 4 (0.3) |  | 15 (1.1) |  | 6 (0.4) |  | 5 (0.4) |  | 2 (0.1) |  | 1 (0.1) |  | 0 (0) |  |
| Chickpea |  | 3 (0.4) |  | 5 (0.7) |  | 3 (0.4) |  | 3 (0.4) |  | 1 (0.1) |  | 0 (0) |  | 0 (0) |  |
| Rice-lentil |  | 7 (0.9) |  | 20 (2.6) |  | 12 (1.6) |  | 10 (1.3) |  | 4 (0.5) |  | 3 (0.4) |  | 0 (0) |  |
| WSB++ |  | 3 (0.4) |  | 12 (1.6) |  | 8 (1.0) |  | 4 (0.5) |  | 1 (0.1) |  | 0 (0) |  | 0 (0) |  |
| 12 mo | 6.8 |  | 0.0000 |  | 0.0000 |  | 0.0000 |  | 0.0000 |  | 0.0000 |  | 0.0000 |  |  |
| CFC-only |  | 45 (3.4) |  | 112 (8.5) |  | 76 (5.8) |  | 58 (4.4) |  | 36 (2.7) |  | 24 (1.8) |  | 0 (0) |  |
| Plumpy’doz |  | 4 (0.3) |  | 17 (1.2) |  | 8 (0.6) |  | 7 (0.5) |  | 4 (0.3) |  | 0 (0) |  | 0 (0) |  |
| Chickpea |  | 0 (0) |  | 3 (0.4) |  | 1 (0.1) |  | 1 (0.1) |  | 0 (0) |  | 0 (0) |  | 0 (0) |  |
| Rice-lentil |  | 3 (0.4) |  | 6 (0.8) |  | 3 (0.4) |  | 3 (0.4) |  | 2 (0.3) |  | 1 (0.1) |  | 0 (0) |  |
| WSB++ |  | 0 (0) |  | 4 (0.5) |  | 1 (0.1) |  | 1 (0.1) |  | 0 (0) |  | 0 (0) |  | 0 (0) |  |
| 15 mo | 7.1 |  | 0.0000 |  | 0.0000 |  | 0.0000 |  | 0.0000 |  | 0.0000 |  | 0.0000 |  | 0.2233 |
| CFC-only |  | 41 (3.2) |  | 87 (6.7) |  | 56 (4.3) |  | 46 (3.6) |  | 32 (2.5) |  | 23 (1.8) |  | 2 (0.2) |  |
| Plumpy’doz |  | 5 (0.4) |  | 11 (0.8) |  | 6 (0.4) |  | 5 (0.4) |  | 5 (0.4) |  | 4 (0.3) |  | 0 (0) |  |
| Chickpea |  | 1 (0.1) |  | 7 (0.9) |  | 4 (0.5) |  | 2 (0.3) |  | 1 (0.1) |  | 1 (0.1) |  | 0 (0) |  |
| Rice-lentil |  | 3 (0.4) |  | 5 (0.7) |  | 3 (0.4) |  | 3 (0.4) |  | 3 (0.4) |  | 3 (0.4) |  | 0 (0) |  |
| WSB++ |  | 2 (0.3) |  | 7 (0.9) |  | 5 (0.6) |  | 3 (0.4) |  | 2 (0.3) |  | 1 (0.1) |  | 0 (0) |  |
| 18 mo | 7.5 |  | 0.0000 |  | 0.0000 |  | 0.0000 |  | 0.0000 |  | 0.0000 |  | 0.0000 |  | 0.0019 |
| CFC-only |  | 35 (2.8) |  | 71 (5.6) |  | 53 (4.2) |  | 43 (3.4) |  | 30 (2.4) |  | 23 (1.8) |  | 12 (0.9) |  |
| Plumpy’doz |  | 7 (0.5) |  | 15 (1.1) |  | 9 (0.7) |  | 8 (0.6) |  | 4 (0.3) |  | 1 (0.1) |  | 0 (0) |  |
| Chickpea |  | 5 (0.7) |  | 8 (1.1) |  | 5 (0.7) |  | 5 (0.7) |  | 5 (0.7) |  | 4 (0.5) |  | 2 (0.3) |  |
| Rice-lentil |  | 6 (0.8) |  | 10 (1.3) |  | 9 (1.2) |  | 6 (0.8) |  | 4 (0.5) |  | 3 (0.4) |  | 2 (0.3) |  |
| WSB++ |  | 8 (1.1) |  | 12 (1.6) |  | 10 (1.3) |  | 8 (1.1) |  | 8 (1.1) |  | 5 (0.7) |  | 2 (0.3) |  |
| 24 mo | 8.3 |  | 0.7094 |  | 0.3156 |  | 0.5671 |  | 0.5375 |  | 0.5410 |  | 0.7457 |  | 0.8440 |
| CFC-only |  | 34 (2.8) |  | 57 (4.6) |  | 44 (3.6) |  | 41 (3.3) |  | 30 (2.4) |  | 21 (1.7) |  | 14 (1.1) |  |
| Plumpy’doz |  | 27 (2.2) |  | 43 (3.5) |  | 33 (2.7) |  | 30 (2.4) |  | 23 (1.9) |  | 18 (1.5) |  | 15 (1.2) |  |
| Chickpea |  | 15 (2.2) |  | 24 (3.4) |  | 19 (2.7) |  | 18 (2.6) |  | 12 (1.7) |  | 8 (1.1) |  | 7 (1.0) |  |
| Rice-lentil |  | 14 (2.0) |  | 28 (3.9) |  | 23 (3.2) |  | 21 (3.0) |  | 14 (2.0) |  | 12 (1.7) |  | 7 (1.0) |  |
| WSB++ |  | 14 (1.9) |  | 21 (2.9) |  | 18 (2.5) |  | 16 (2.2) |  | 10 (1.4) |  | 8 (1.1) |  | 5 (0.7) |  |

CFC, child feeding counseling; EAR, Estimated Average Requirement; WSB++, Wheat-Soy Blend.

^1^Energy and protein intakes include reported complementary food intake from a 24-hr recall, reported daily supplement intake, and age-specific estimated breast milk intake. From 6 to 11 months, the supplements were delivered in small quantities of 125 kcal/day and 2.6 g of protein. From 12 to 18 months, the supplements were delivered in medium quantities of 250 kcal/day and 5.2 g of protein. It was not possible to estimate adjusted protein intake for the group receiving PD from 9 to 18 months because information is not available to estimate the DIAAS for this commercially produced product.

^2^Estimated using mean weight of control group infants and children at each age time point.

^3^Chi^2^ p-values are from log binomial regression models with generalized estimating equations to allow for clustering of observations within sectors.

| **Supplemental Table 7**. Prevalence of inadequate protein intakes (DIAAS-adjusted) by age and supplement group among children enrolled in a complementary food supplementation trial in Bangladesh^1^ | | | | | | | | | | | | | | | |
| --- | --- | --- | --- | --- | --- | --- | --- | --- | --- | --- | --- | --- | --- | --- | --- |
|  |  | Assumed breastmilk intake as a % of the WHO 1998 average intake level | | | | | | | | | | | | | |
| Age | EAR, g/d^2^ | 100% | | 80% | | 90% | | 95% | | 105% | | 110% | | 120% | |
|  |  | Inadequate intake, % (n) | Chi^2^ p-value^3^ | Inadequate intake, % (n) | Chi^2^ p-value^3^ | Inadequate intake, % (n) | Chi^2^ p-value^3^ | Inadequate intake, % (n) | Chi^2^ p-value^3^ | Inadequate intake, % (n) | Chi^2^ p-value^3^ | Inadequate intake, % (n) | Chi^2^ p-value^3^ | Inadequate intake, % (n) | Chi^2^ p-value^3^ |
| 6 mo | 10.3 |  | 0.8560 |  | 0.4788 |  | 0.7469 |  | 0.7831 |  | 0.6148 |  | 0.7670 |  | 0.7335 |
| CFC-only |  | 903 (82.5) |  | 969 (88.5) |  | 936 (85.5) |  | 917 (83.7) |  | 880 (80.4) |  | 853 (77.9) |  | 777 (71.0) |  |
| Plumpy’doz |  | 819 (81.3) |  | 887 (88.1) |  | 858 (85.2) |  | 839 (83.3) |  | 789 (78.4) |  | 767 (76.2) |  | 697 (69.2) |  |
| Chickpea |  | 490 (80.3) |  | 524 (85.9) |  | 509 (83.4) |  | 501 (82.1) |  | 471 (77.2) |  | 462 (75.7) |  | 425 (69.7) |  |
| Rice-lentil |  | 456 (81.7) |  | 493 (88.4) |  | 478 (85.7) |  | 471 (84.4) |  | 442 (79.2) |  | 432 (77.4) |  | 400 (71.7) |  |
| WSB++ |  | 480 (82.1) |  | 521 (89.1) |  | 503 (86.0) |  | 495 (84.6) |  | 462 (79.0) |  | 443 (75.7) |  | 402 (68.7) |  |
| 9 mo | 7.4 |  | 0.0000 |  | 0.0000 |  | 0.0000 |  | 0.0000 |  | 0.0000 |  | 0.0000 |  |  |
| CFC-only |  | 159 (12.3) |  | 397 (30.7) |  | 291 (22.5) |  | 228 (17.6) |  | 91 (7.0) |  | 40 (3.1) |  | 0 (0) |  |
| Plumpy’doz |  | - |  | - |  | - |  | - |  | - |  | - |  | - |  |
| Chickpea |  | 3 (0.4) |  | 10 (1.3) |  | 6 (0.8) |  | 5 (0.6) |  | 1 (0.1) |  | 1 (0.1) |  | 0 (0) |  |
| Rice-lentil |  | 12 (1.5) |  | 28 (3.6) |  | 21 (2.7) |  | 14 (1.8) |  | 8 (1.0) |  | 4 (0.5) |  | 0 (0) |  |
| WSB++ |  | 5 (0.6) |  | 19 (2.4) |  | 10 (1.3) |  | 7 (0.9) |  | 3 (0.4) |  | 1 (0.1) |  | 0 (0) |  |
| 12 mo | 6.8 |  | 0.0000 |  | 0.0000 |  | 0.0000 |  | 0.0000 |  | 0.0000 |  | 0.0000 |  |  |
| CFC-only |  | 56 (4.3) |  | 187 (14.2) |  | 111 (8.4) |  | 86 (6.5) |  | 42 (3.2) |  | 23 (1.7) |  | 0 (0) |  |
| Plumpy’doz |  | - |  | - |  | - |  | - |  | - |  | - |  | - |  |
| Chickpea |  | 1 (0.1) |  | 3 (0.4) |  | 3 (0.4) |  | 2 (0.3) |  | 1 (0.1) |  | 0 (0) |  | 0 (0) |  |
| Rice-lentil |  | 4 (0.5) |  | 9 (1.2) |  | 4 (0.5) |  | 4 (0.5) |  | 3 (0.4) |  | 1 (0.1) |  | 0 (0) |  |
| WSB++ |  | 1 (0.1) |  | 6 (0.8) |  | 2 (0.3) |  | 1 (0.1) |  | 0 (0) |  | 0 (0) |  | 0 (0) |  |
| 15 mo | 7.1 |  | 0.0000 |  | 0.0000 |  | 0.0000 |  | 0.0000 |  | 0.0000 |  | 0.0000 |  |  |
| CFC-only |  | 51 (4.0) |  | 143 (11.1) |  | 96 (7.4) |  | 65 (5.0) |  | 36 (2.8) |  | 28 (2.2) |  | 0 (0) |  |
| Plumpy’doz |  | - |  | - |  | - |  | - |  | - |  | - |  | - |  |
| Chickpea |  | 2 (0.3) |  | 10 (1.3) |  | 6 (0.8) |  | 4 (0.5) |  | 2 (0.3) |  | 1 (0.1) |  | 0 (0) |  |
| Rice-lentil |  | 4 (0.5) |  | 5 (0.7) |  | 5 (0.7) |  | 5 (0.7) |  | 3 (0.4) |  | 3 (0.4) |  | 0 (0) |  |
| WSB++ |  | 5 (0.6) |  | 8 (1.0) |  | 7 (0.9) |  | 6 (0.8) |  | 3 (0.4) |  | 2 (0.3) |  | 0 (0) |  |
| 18 mo | 7.5 |  | 0.0000 |  | 0.0000 |  | 0.0000 |  | 0.0000 |  | 0.0000 |  | 0.0009 |  | 0.0555 |
| CFC-only |  | 59 (4.7) |  | 100 (7.9) |  | 75 (5.9) |  | 66 (5.2) |  | 41 (3.2) |  | 28 (2.2) |  | 13 (1.0) |  |
| Plumpy’doz |  | - |  | - |  | - |  | - |  | - |  | - |  | - |  |
| Chickpea |  | 5 (0.7) |  | 20 (2.7) |  | 7 (0.9) |  | 5 (0.7) |  | 5 (0.7) |  | 4 (0.5) |  | 3 (0.4) |  |
| Rice-lentil |  | 9 (1.2) |  | 15 (2.0) |  | 12 (1.6) |  | 9 (1.2) |  | 6 (0.8) |  | 5 (0.7) |  | 2 (0.3) |  |
| WSB++ |  | 10 (1.3) |  | 24 (3.2) |  | 18 (2.4) |  | 13 (1.7) |  | 7 (0.9) |  | 6 (0.8) |  | 2 (0.3) |  |
| 24 mo | 8.3 |  | 0.2612 |  | 0.7475 |  | 0.4813 |  | 0.1368 |  | 0.5796 |  | 0.7880 |  | 0.9741 |
| CFC-only |  | 57 (4.6) |  | 90 (7.3) |  | 76 (6.2) |  | 71 (5.8) |  | 44 (3.6) |  | 34 (2.8) |  | 19 (1.5) |  |
| Plumpy’doz |  | 43 (3.5) |  | 77 (6.2) |  | 57 (4.6) |  | 50 (4.0) |  | 35 (2.8) |  | 28 (2.3) |  | 18 (1.5) |  |
| Chickpea |  | 22 (3.2) |  | 52 (7.5) |  | 37 (5.3) |  | 27 (3.9) |  | 19 (2.7) |  | 15 (2.2) |  | 9 (1.3) |  |
| Rice-lentil |  | 25 (3.5) |  | 46 (6.5) |  | 38 (5.4) |  | 32 (4.5) |  | 21 (3.0) |  | 16 (2.3) |  | 11 (1.5) |  |
| WSB++ |  | 21 (2.9) |  | 47 (6.4) |  | 35 (4.8) |  | 27 (3.7) |  | 17 (2.3) |  | 14 (1.9) |  | 9 (1.2) |  |

CFC, child feeding counseling; EAR, Estimated Average Requirement; WSB++, Wheat-Soy Blend.

^1^Energy and protein intakes include reported complementary food intake from a 24-hr recall, reported daily supplement intake, and age-specific estimated breast milk intake. From 6 to 11 months, the supplements were delivered in small quantities of 125 kcal/day and 2.6 g of protein. From 12 to 18 months, the supplements were delivered in medium quantities of 250 kcal/day and 5.2 g of protein. Using the digestible indispensable amino acid score (DIAAS) to adjust for protein quality. It was not possible to estimate adjusted protein intake for the group receiving PD from 9 to 18 months because information is not available to estimate the DIAAS for this commercially produced product.

^2^Estimated using mean weight of control group infants and children at each age time point.

^3^Chi^2^ p-values are from log binomial regression models with generalized estimating equations to allow for clustering of observations within sectors.

| **Supplemental Table 8**. Prevalence of inadequate protein intakes (DIAAS and infection adjusted) by age and supplement group among children enrolled in a complementary food supplementation trial in Bangladesh^1^ | | | | | | | | | | | | | | |  |
| --- | --- | --- | --- | --- | --- | --- | --- | --- | --- | --- | --- | --- | --- | --- | --- |
|  |  | Assumed breastmilk intake as a % of the WHO 1998 average intake level | | | | | | | | | | | | | |
| Age | EAR, g/d^2^ | 100% | | 80% | | 90% | | 95% | | 105% | | 110% | | 120% | |
|  |  | Inadequate intake, % (n) | Chi^2^ p-value^3^ | Inadequate intake, % (n) | Chi^2^ p-value^3^ | Inadequate intake, % (n) | Chi^2^ p-value^3^ | Inadequate intake, % (n) | Chi^2^ p-value^3^ | Inadequate intake, % (n) | Chi^2^ p-value^3^ | Inadequate intake, % (n) | Chi^2^ p-value^3^ | Inadequate intake, % (n) | Chi^2^ p-value^3^ |
| 6 mo | 10.3 |  | 0.5207 |  | 0.3615 |  | 0.4768 |  | 0.4331 |  | 0.6164 |  | 0.8645 |  | 0.6663 |
| CFC-only |  | 958 (87.5) |  | 998 (91.1) |  | 980 (89.5) |  | 972 (88.8) |  | 942 (86.0) |  | 922 (84.2) |  | 886 (80.9) |  |
| Plumpy’doz |  | 881 (87.5) |  | 913 (90.7) |  | 904 (89.8) |  | 891 (88.5) |  | 861 (85.5) |  | 845 (83.9) |  | 796 (79.0) |  |
| Chickpea |  | 517 (84.8) |  | 543 (89.0) |  | 535 (87.7) |  | 527 (86.4) |  | 509 (83.4) |  | 506 (83.0) |  | 476 (78.0) |  |
| Rice-lentil |  | 484 (86.7) |  | 512 (91.8) |  | 505 (90.5) |  | 497 (89.1) |  | 481 (86.2) |  | 473 (84.8) |  | 446 (79.9) |  |
| WSB++ |  | 511 (87.4) |  | 539 (92.1) |  | 530 (90.6) |  | 525 (89.7) |  | 503 (86.0) |  | 498 (85.1) |  | 463 (79.1) |  |
| 9 mo | 7.4 |  | 0.0000 |  | 0.0000 |  | 0.0000 |  | 0.0000 |  | 0.0000 |  | 0.0000 |  | 0.0000 |
| CFC-only |  | 324 (25.1) |  | 523 (40.4) |  | 425 (32.9) |  | 379 (29.3) |  | 264 (20.4) |  | 198 (15.3) |  | 61 (4.7) |  |
| Plumpy’doz |  | - |  | - |  | - |  | - |  | - |  | - |  | - |  |
| Chickpea |  | 8 (1.0) |  | 17 (2.2) |  | 11 (1.4) |  | 8 (1.0) |  | 6 (0.8) |  | 3 (0.4) |  | 1 (0.1) |  |
| Rice-lentil |  | 22 (2.8) |  | 73 (9.4) |  | 37 (4.8) |  | 26 (3.4) |  | 18 (2.3) |  | 12 (1.5) |  | 6 (0.8) |  |
| WSB++ |  | 11 (1.4) |  | 25 (3.2) |  | 19 (2.4) |  | 16 (2.1) |  | 9 (1.2) |  | 6 (0.8) |  | 1 (0.1) |  |
| 12 mo | 6.8 |  | 0.0000 |  | 0.0000 |  | 0.0000 |  | 0.0000 |  | 0.0000 |  | 0.0000 |  | 0.0000 |
| CFC-only |  | 134 (10.2) |  | 278 (21.1) |  | 206 (15.7) |  | 171 (13.0) |  | 101 (7.7) |  | 71 (5.4) |  | 33 (2.5) |  |
| Plumpy’doz |  | - |  | - |  | - |  | - |  | - |  | - |  | - |  |
| Chickpea |  | 3 (0.4) |  | 6 (0.8) |  | 4 (0.5) |  | 3 (0.4) |  | 3 (0.4) |  | 2 (0.3) |  | 0 (0) |  |
| Rice-lentil |  | 6 (0.8) |  | 21 (2.7) |  | 12 (1.5) |  | 7 (0.9) |  | 4 (0.5) |  | 4 (0.5) |  | 2 (0.3) |  |
| WSB++ |  | 3 (0.4) |  | 9 (1.1) |  | 8 (1.0) |  | 4 (0.5) |  | 2 (0.3) |  | 1 (0.1) |  | 0 (0) |  |
| 15 mo | 7.1 |  | 0.0000 |  | 0.0000 |  | 0.0000 |  | 0.0000 |  | 0.0000 |  | 0.0000 |  | 0.0000 |
| CFC-only |  | 115 (8.9) |  | 221 (17.1) |  | 160 (12.4) |  | 135 (10.5) |  | 88 (6.8) |  | 61 (4.7) |  | 33 (2.6) |  |
| Plumpy’doz |  | - |  | - |  | - |  | - |  | - |  | - |  | - |  |
| Chickpea |  | 8 (1.0) |  | 10 (1.3) |  | 10 (1.3) |  | 8 (1.0) |  | 6 (0.8) |  | 4 (0.5) |  | 2 (0.3) |  |
| Rice-lentil |  | 5 (0.7) |  | 12 (1.6) |  | 6 (0.8) |  | 5 (0.7) |  | 5 (0.7) |  | 5 (0.7) |  | 3 (0.4) |  |
| WSB++ |  | 7 (0.9) |  | 13 (1.7) |  | 10 (1.3) |  | 8 (1.0) |  | 7 (0.9) |  | 6 (0.8) |  | 2 (0.3) |  |
| 18 mo | 7.5 |  | 0.0000 |  | 0.0000 |  | 0.0000 |  | 0.0000 |  | 0.0000 |  | 0.0000 |  | 0.0000 |
| CFC-only |  | 88 (7.0) |  | 158 (12.5) |  | 119 (9.4) |  | 99 (7.8) |  | 72 (5.7) |  | 65 (5.1) |  | 36 (2.8) |  |
| Plumpy’doz |  | - |  | - |  | - |  | - |  | - |  | - |  | - |  |
| Chickpea |  | 13 (1.7) |  | 29 (3.8) |  | 22 (2.9) |  | 16 (2.1) |  | 6 (0.8) |  | 5 (0.7) |  | 5 (0.7) |  |
| Rice-lentil |  | 14 (1.9) |  | 32 (4.2) |  | 19 (2.5) |  | 15 (2.0) |  | 11 (1.5) |  | 9 (1.2) |  | 6 (0.8) |  |
| WSB++ |  | 19 (2.5) |  | 34 (4.5) |  | 27 (3.6) |  | 23 (3.0) |  | 17 (2.2) |  | 12 (1.6) |  | 7 (0.9) |  |
| 24 mo | 8.3 |  | 0.4624 |  | 0.9147 |  | 0.6940 |  | 0.8493 |  | 0.4655 |  | 0.1450 |  | 0.4381 |
| CFC-only |  | 86 (7.0) |  | 127 (10.3) |  | 101 (8.2) |  | 91 (7.4) |  | 77 (6.2) |  | 72 (5.8) |  | 46 (3.7) |  |
| Plumpy’doz |  | 65 (5.2) |  | 117 (9.4) |  | 91 (7.3) |  | 80 (6.5) |  | 58 (4.7) |  | 50 (4.0) |  | 35 (2.8) |  |
| Chickpea |  | 43 (6.2) |  | 70 (10.0) |  | 62 (8.9) |  | 52 (7.5) |  | 37 (5.3) |  | 28 (4.0) |  | 19 (2.7) |  |
| Rice-lentil |  | 42 (5.9) |  | 75 (10.6) |  | 63 (8.9) |  | 46 (6.5) |  | 39 (5.5) |  | 33 (4.6) |  | 21 (3.0) |  |
| WSB++ |  | 41 (5.6) |  | 70 (9.5) |  | 57 (7.8) |  | 50 (6.8) |  | 35 (4.8) |  | 28 (3.8) |  | 17 (2.3) |  |

CFC, child feeding counseling; EAR, Estimated Average Requirement; WSB++, Wheat-Soy Blend.

^1^Energy and protein intakes include reported complementary food intake from a 24-hr recall, reported daily supplement intake, and age-specific estimated breast milk intake. From 6 to 11 months, the supplements were delivered in small quantities of 125 kcal/day and 2.6 g of protein. From 12 to 18 months, the supplements were delivered in medium quantities of 250 kcal/day and 5.2 g of protein. Using the digestible indispensable amino acid score (DIAAS) to adjust for protein quality. EAR adjusted for DIAAS plus inadequate energy intake and infections assuming 10% additional protein requirement due to dietary energy deficiency, plus 10% increase in protein requirement during each day of illness, 7 days per episode, 5.3 episodes per year, or 11.1% increase in protein requirement relative to the EAR. It was not possible to estimate adjusted protein intake for the group receiving PD from 9 to 18 months because information is not available to estimate the DIAAS for this commercially produced product.

^2^Estimated using mean weight of control group infants and children at each age time point.

^3^Chi^2^ p-values are from log binomial regression models with generalized estimating equations to allow for clustering of observations within sectors.
